# Comprehensive analysis of promoter-proximal RNA polymerase II pausing across mammalian cell types

**DOI:** 10.1186/s13059-016-0984-2

**Published:** 2016-06-03

**Authors:** Daniel S. Day, Bing Zhang, Sean M. Stevens, Francesco Ferrari, Erica N. Larschan, Peter J. Park, William T. Pu

**Affiliations:** Department of Biomedical Informatics, Harvard Medical School, Boston, MA 02115 USA; Harvard/MIT Division of Health Sciences and Technology, Cambridge, MA 02139 USA; Department of Cardiology, Boston Children’s Hospital, Boston, MA 02115 USA; Shanghai Center for Systems Biomedicine, Shanghai Jiao Tong University, Shanghai, 200240 China; Department of Molecular Biology, Cellular Biology and Biochemistry, Brown University, Providence, RI 02912 USA; Harvard Stem Cell Institute, Cambridge, MA 02138 USA

## Abstract

**Background:**

For many genes, RNA polymerase II stably pauses before transitioning to productive elongation. Although polymerase II pausing has been shown to be a mechanism for regulating transcriptional activation, the extent to which it is involved in control of mammalian gene expression and its relationship to chromatin structure remain poorly understood.

**Results:**

Here, we analyze 85 RNA polymerase II chromatin immunoprecipitation (ChIP)-sequencing experiments from 35 different murine and human samples, as well as related genome-wide datasets, to gain new insights into the relationship between polymerase II pausing and gene regulation. Across cell and tissue types, paused genes (pausing index > 2) comprise approximately 60 % of expressed genes and are repeatedly associated with specific biological functions. Paused genes also have lower cell-to-cell expression variability. Increased pausing has a non-linear effect on gene expression levels, with moderately paused genes being expressed more highly than other paused genes. The highest gene expression levels are often achieved through a novel pause-release mechanism driven by high polymerase II initiation. In three datasets examining the impact of extracellular signals, genes responsive to stimulus have slightly lower pausing index on average than non-responsive genes, and rapid gene activation is linked to conditional pause-release. Both chromatin structure and local sequence composition near the transcription start site influence pausing, with divergent features between mammals and *Drosophila*. Most notably, in mammals pausing is positively correlated with histone H2A.Z occupancy at promoters.

**Conclusions:**

Our results provide new insights into the contribution of RNA polymerase II pausing in mammalian gene regulation and chromatin structure.

**Electronic supplementary material:**

The online version of this article (doi:10.1186/s13059-016-0984-2) contains supplementary material, which is available to authorized users.

## Background

The initiation of RNA polymerase II (RNAP2) is a highly regulated process in mammalian cells [1, 2]. A wide variety of proteins, from general transcription factors to chromatin remodelers, interact with and mediate the activity of RNAP2, as it binds a promoter, initiates transcription, and begins to elongate. After RNAP2 enters early elongation, it pauses about 20–60 bp downstream from the transcriptional start site (TSS) [1–3]. This promoter-proximal pausing of RNAP2 (“RNAP2 pausing”) is increasingly recognized as an important step in regulating gene expression [1]. RNAP2 pausing is induced by the binding of negative elongation factor (NELF) and DRB-sensitivity inducing factor (DSIF) to RNAP2. Paused RNAP2 remains stable until it is released from its paused state by positive transcription elongation factor b (P-TEFb), a complex that includes cyclin-dependent kinase 9 (CDK9). P-TEFb triggers paused RNAP2 to enter productive elongation by phosphorylating serine 2 on the C-terminal domain of RNAP2 and removing NELF [1, 2]. For at least a subset of metazoan genes, RNAP2 pausing is a rate-limiting step in transcription and this additional regulatory layer represents a paradigm shift in our understanding of gene transcriptional regulation, since RNAP2 initiation has long been viewed as the main determinant of gene transcription [1].

However, the relationship between RNAP2 pausing and the regulation of chromatin structure and gene expression is poorly understood [1]. Several studies have suggested that RNAP2 pausing-release mediates rapid gene expression changes in response to external stimuli [1, 4, 5]. But, in fact, many other genes in addition to the rapid response genes display significant promoter-proximal RNAP2 accumulation [1, 6–8], suggesting additional regulatory roles for stable RNAP2 pausing. On the other hand, recent reports suggest that stably paused RNAP2 shapes the chromatin structure of promoters [9–11], but the extent that RNAP2 pausing regulates chromatin remodeling across mammalian cell types has not been carefully examined. With advances in profiling various histone modifications, chromatin-bound proteins, and genome-wide expression levels in an unbiased manner, a broad, in-depth analysis of such data would better highlight and refine the role RNAP2 pausing has on regulating gene expression and chromatin structure in mammals.

In order to better refine the roles of RNAP2 pausing on regulating gene expression and chromatin structure, we performed a comprehensive analysis of promoter-proximal RNAP2 accumulation across multiple mammalian cell types using publicly available RNAP2 chromatin immunoprecipitation (ChIP)-sequencing (ChIP-seq) and related datasets (e.g. from the ENCODE project). This allowed us to generalize previous observations from specific cell types as well as to have a greater power to detect correlation structure between promoter-proximal RNAP2 accumulation and gene expression or chromatin structure. We found that, for many genes, there is recurrent enrichment or depletion of promoter-proximal RNAP2 across cell types, and that the DNA sequences at promoters are likely to contribute to this phenomenon. While promoter-proximal RNAP2 accumulation did not strongly predict steady-state gene expression levels, it lowered gene expression variability across a cell population and mediated rapid gene upregulation in response to extracellular cues. Finally, we found that the density of H2A.Z at promoters correlated positively with higher amounts of paused RNAP2 in mammalian cells, contrary to the negative correlation observed in *Drosophila* [12]. Overall, our computational analysis provides new insights into the contribution of RNAP2 pausing to global regulation of gene expression in mammalian cells.

## Results

### Characterization of RNAP2 pausing across multiple cell types

**Fig. 1:**
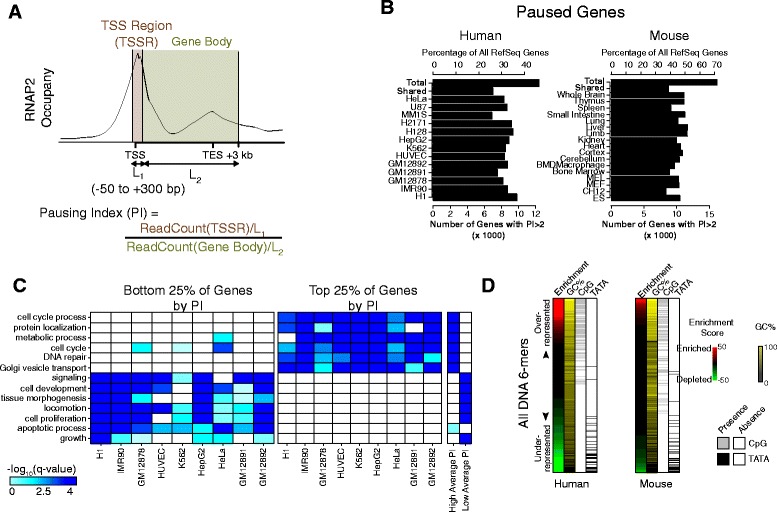
Overview of paused genes across multiple human and mouse cell types. **a** Estimation of a gene’s “pausing index” (PI) from RNAP2 ChIP-seq data. **b** Occurrence of paused genes across cell types. The frequency of paused genes (PI ≥2) was similar in diverse human and mouse cell types. **c** Functional annotations enriched among the most or least paused genes in human cell lines. The top quartile of genes by PI rank had similar GO biological process term enrichment across both normal and cancer cell types, as did the bottom quartile. Similar enrichments were observed when considering genes with pausing greater than (“high average PI”) or less than (“low average PI”) the median PI across all cell types. **d** Sequence composition analysis of gene promoters. All DNA 6-mers were tested for enrichment in human paused promoters versus non-paused promoters. Each 6-mer was ranked by its enrichment score (see “Methods”). Human paused promoters were over-represented for 6-mers with high GC and CpG content and depleted for the TATA motif

We analyzed RNAP2 pausing at each gene based on its “Pausing Index” (PI; also referred to as “Traveling Ratio”) [1, 7, 8, 13, 14]. PI has been used previously as a proxy for the level of promoter-proximal RNAP2 pausing at a gene [8, 13–15] and is defined as the ratio between the amount of RNAP2 that accumulates near the promoter (predominantly paused RNAP2 [9]) and the amount of RNAP2 found in the remainder of the gene (predominately elongating RNAP2), as shown in Fig. 1a. To measure the occupancy of RNAP2, we used RNAP2 ChIP-seq data. Although RNAP2 ChIP-seq is less sensitive than other techniques designed specifically for measuring paused RNAP2, such as GRO-seq (global run-on sequencing) [15] or PRO-seq (precise run-on sequencing) [3], a comparison between GRO-seq and RNAP2 ChIP-seq data suggested that most signals observed in RNAP2 ChIP-seq data come from transcriptionally engaged RNAP2 [9], supporting their use for measuring differences in RNAP2 pausing. Importantly, a large amount of RNAP2 ChIP-seq data is publicly available, allowing us to analyze RNAP2 pausing across a wide range of human and mouse cell types.

Operationally, we estimated a PI as the ratio of normalized RNAP2 ChIP-seq read density within the TSS region (TSSR, –50 to +300 bp around TSS) to that in the gene body (TSS + 300 bp to +3 kb past the annotated transcriptional end site (TES); Fig. 1a and Additional file 1: Figure S1; see “Methods”). To remove noise from genes with low transcriptional activity, those genes with RNAP2 and H3K4me3 TSSR density below specified thresholds were excluded from further analyses in that cell type (see “Methods”). For genes with multiple annotated TSSs, we assigned the TSS having the strongest H3K4me3 signal as its primary TSS (see “Methods”). Our estimated PI values correlated well across biological replicates even when different RNAP2 antibodies were used (Additional file 1: Figure S2A, B). Furthermore, two independent markers of RNAP2 elongation, H3K36me3 and RNAP2 phosphorylated on serine 2 of its C-terminal repeat domain (RNAP2 pS2), strongly correlated with our gene body RNAP2 density estimates (Additional file 1: Figure S2C, D), indicating that we quantified elongating RNAP2 accurately.

We used PI to examine how RNAP2 pausing at a gene relates to its other properties, such as biological function, expression level, and local chromatin structure. We first assessed the prevalence of pausing across 64 human and 24 mouse RNAP2 ChIP-seq datasets spanning multiple cell lines and tissue types (Additional file 2: Table S1). We considered a gene to be paused if PI >2 (i.e. at least twofold more paused RNAP2 compared to elongating RNAP2), as was done in previous studies [8, 13, 14]. At this threshold, RNAP2 pausing was widespread: genes with a PI >2 accounted for 33 ± 4 % and 46 ± 7 % of RefSeq annotated genes for the human and mouse samples, respectively (Fig. 1b and Additional file 1: Figure S3A). The paused genes were also consistent across cell types: among the genes with a PI >2 in any cell type, more than half had a PI >2 in >75 % of cell types, both for human and mouse (Additional file 1: Figure S3B). Moreover, the underlying cell type or state (e.g. of embryonic, adult, or cancerous origin) appeared to have little impact on whether a gene was paused at steady-state.

Given the consistency of how promoter-proximal paused RNAP2 was deployed across cell types, we wanted to understand whether any biological functions were enriched with genes that tended to have more or less paused RNAP2. Accordingly, we searched across cell types for biological functions commonly enriched in genes with higher or lower amount of pausing, defined as the top or bottom 25 % of genes by PI. Among the Gene Ontology (GO) biological process (BP) terms found in each cell type (Fig. 1c, Additional file 1: Figure S3C, and Additional file 3: Table S2; see “Methods”), many were enriched across many cell types. Genes with high PI were enriched for GO terms involving cellular metabolism, DNA repair, protein localization, and cell cycle; those with low PI were enriched for developmental, apoptosis, and cell signaling terms (Fig. 1c, Additional file 1: Figure S3C, and Additional file 3: Table S2). To ensure robustness of our results, we repeated the enrichment analysis based on the rank of each gene (median PI across cell types) and found that many GO terms were maintained. These analyses indicate that some functional classes of genes share RNAP2 pausing properties across cell types, perhaps reflecting shared gene regulatory mechanisms.

This consistency across diverse cell types led us to consider whether common genomic features may regulate this phenomenon. In particular, we focused on identifying DNA sequence patterns enriched in promoters (+/–500 bp of the TSS) of paused genes. Genes with a PI >2 in at least one cell type (see “Methods”) tended to have a significantly higher promoter GC and CpG content relative to unpaused genes (PI <2 in all cell types examined; Additional file 1: Figure S4A), consistent with a previous report in a single cell type [15]. Motif analysis revealed that paused promoters were enriched for motifs with high GC and CpG content and depleted for the TATA motif (Fig. 1d and Additional file 1: Figure S4B). Together, these results suggest that the composition of promoter sequence influences promoter-proximal RNAP2 pausing across a diverse set of mammalian cell types.

### Differential levels of paused RNAP2 dampen cell-to-cell expression variability

**Fig. 2:**
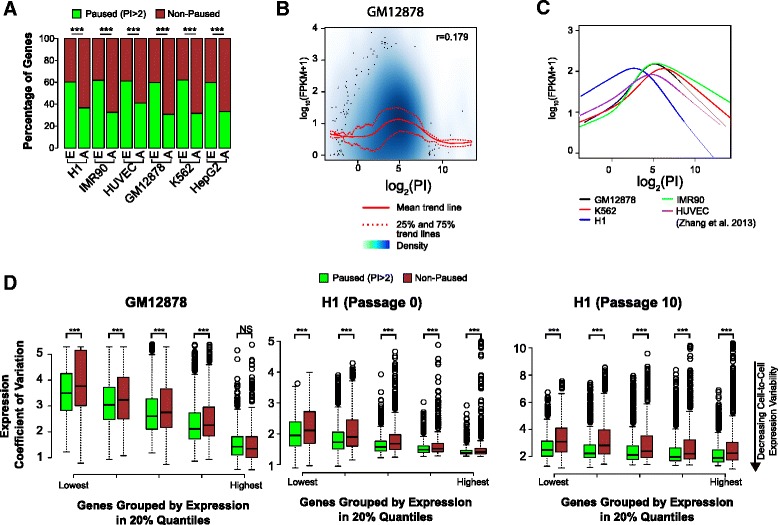
Paused genes have lower cell-to-cell expression variability than non-paused genes. **a** Paused genes comprised the majority of expressed (FPKM >1) genes in each cell type. E, expressed genes (FPKM >1); A, All Refseq genes. (***p <0.001, Mann–Whitney *U* test). **b** “Hill-shaped” relationship between gene expression and PI in GM12878. We found no linear correlation between the PI and gene expression levels. The broadest range and strongest level of gene expression occurred at intermediate PI values, while extreme PI values were associated with reduced gene expression. **c** Similar “hill-shaped” relationships were observed in the four other cell lines examined, with the hill peak occurring within a similar PI range for 4/5 cell lines. **d** Using single cell RNA-seq data for GM12878 [18] and H1 [19], we analyzed the effect of RNAP2 pausing on cell-to-cell gene expression variability. We measured the coefficient of variation (standard deviation/mean) of gene expression across individual cells, stratified by gene expression quintiles based on the single-cell population wide mean expression level. We then compared between paused and non-paused genes. For nearly all expression quintiles in both cell lines, paused genes had lower coefficient of variation on average, suggesting that at the same expression level RNAP2 pausing dampens expression variability. (***p <0.001, NS, not significant; Mann–Whitney *U* test.)

We analyzed the relationship between the degree of RNAP2 pausing at a gene and its transcript levels by comparing PI with RNA-sequencing (RNA-seq) measurements across five mammalian cell lines (see “Methods”). First, in each cell type, the majority of expressed genes (FPKM >1) were paused (Fig. 2a), supporting the idea that genes with paused RNAP2 are often expressed [1]. Next, whereas the density of total RNAP2 binding across the whole gene is well correlated with its gene expression level (for GM12878, Spearman correlation r = 0.531), PI was only weakly correlated (Spearman r = 0.179). Using a cubic smoothing spline to fit the data, we observed a “hill-shaped” trend between PI and gene expression, with genes with a high or low PI having a lower average expression than those with an intermediate PI, across all five cell types (Fig. 2b, c and Additional file 1: Figure S5). This weak correlation between PI and expression level is consistent with prior studies in a single cell type [14, 16] and the non-linear relationship suggests that too little release of paused RNAP2 (high PI) or too little promoter-proximal RNAP2 accumulation (low PI) results in lower expression level.

Since the PI of a gene was only weakly predictive of its expression level, we hypothesized that RNAP2 pausing may influence other aspects of gene expression regulation. Notably, it has been suggested that stably paused RNAP2 reduces gene expression variability across individual cells in a population caused by fluctuations in RNAP2 promoter binding [2]. FISH studies of selected genes in *Drosophila* embryos supported this hypothesis [17]. To test this hypothesis computationally, we examined single-cell RNA-seq data from GM12878 (EBV-immortalized B-cells) [18] and H1 (human embryonic stem cells) [19]. To measure variability in expression, we computed the coefficient of variation (CV; ratio of standard deviation to the mean) in paused and non-paused expressed genes, with “expressed” defined as mean FPKM >1 across cells in each sample. Dividing the genes into five quantiles according to their mean expression level, we found that paused genes had lower expression CV in all but the highest expression quantile (Fig. 2d), even as expression CV decreased with increased expression levels as previously shown [20]. We note that within each quantile, the mean population-wide gene expression was not significantly different between paused and non-paused groups (Additional file 1: Figure S6). Thus, our analysis suggests that paused RNAP2 stabilizes gene expression level across individual cells in a population.

### Contribution of RNAP2 pause-release to changes in gene expression

**Fig. 3:**
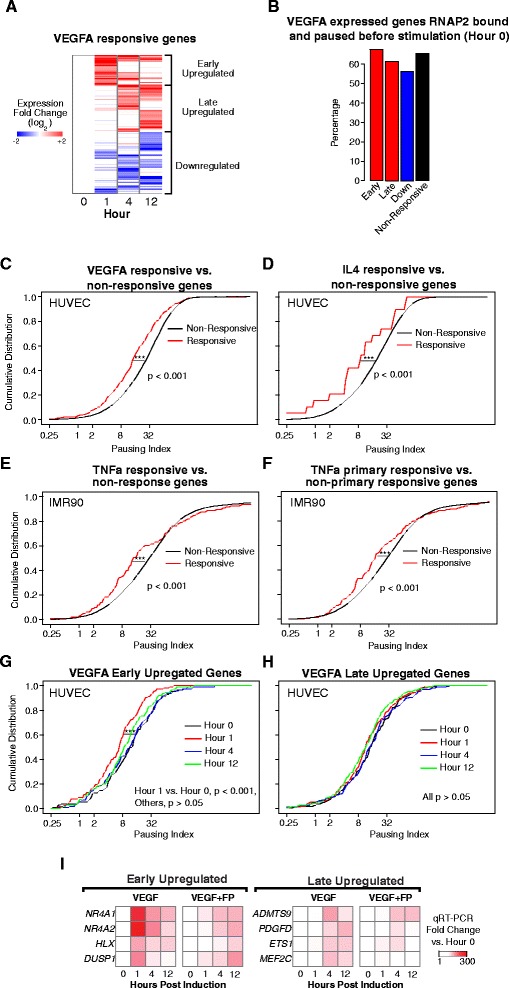
RNAP2 pause-release in signal-induced gene expression. **a** VEGFA-stimulated changes in gene expression in HUVECs [21]. VEGFA-responsive genes were clustered into three groups by the temporal pattern of their VEGFA-induced expression change (see “Methods”). **b** Both the responsive and non-responsive (genes expressed during the time course without a significant change) gene sets had RNAP2 pre-loaded and paused prior to VEGFA-stimulation. **c**–**f** Genes responsive (*red line*) to VEGF (**c**), IL4 (**d**), or TNFα (**e**, **f**) overall had lower PI compared to genes non-responsive (*black line*) to each stimulus. “Primary responsive genes” (**f**) were defined as those that respond in the presence of the protein synthesis inhibitor cycloheximide (***p <0.001; Mann–Whitney *U* test.) **g**, **h** VEGFA rapidly induced overall pause-release among early upregulated genes (**g**) but not late upregulated genes (**h**). **i** Pause-release is required for rapid signal-induced gene expression. Treatment of HUVECs with pause-release inhibitor FP markedly attenuated VEGFA activation of early upregulated genes. FP had more moderate effects on VEGFA activation of late upregulated genes

It has been proposed that a key role for RNAP2 pausing is to poise some genes for rapid, stimulus-induced activation [1, 4, 5]. To investigate the applicability of this transcriptional regulatory mechanism to other physiological cell responses, we analyzed several mammalian RNAP2 ChIP-seq and matched RNA-seq datasets that characterize responses to cellular signals. Using our recent vascular endothelial growth factor A (VEGFA) stimulation time-course data [21], we tested whether VEGFA regulates angiogenesis-response in HUVECs through changes in RNAP2 pause-release. We grouped genes differentially expressed in response to VEGFA (greater than absolute twofold change in expression compared to pre-stimulus) into early upregulated, late upregulated, and downregulated gene sets (Fig. 3a; see “Methods”). All remaining expressed genes were categorized as non-responsive (see “Methods”).

Similar to the cell lines shown in Fig. 2a, but contrary to LPS-responsive *Drosophila* S2 cells [6], the majority of genes in each responsive set were paused prior to stimulation (Fig. 3b). Surprisingly, we discovered that, prior to stimulation, each responsive gene set had a different distribution of PIs than non-responsive genes, with generally lower PIs (*p* <0.001, Mann–Whitney *U* test; Fig. 3c and Additional file 1: Figure S7A). To determine if this observation extended to other stimuli and cell types, we performed two additional analyses. First, we identified genes that were responsive to IL-4 stimulation of HUVEC cells from a microarray expression dataset [22] (see “Methods”). As with VEGFA responsive genes, these IL-4 responsive genes also had a lower distribution of PIs than IL-4 non-responsive genes in unstimulated HUVECs, based on our baseline (Hour 0) HUVEC RNAP2 ChIP-seq data (*p* <0.001, Mann–Whitney *U* test; Fig. 3d). Second, we tested whether this observation extended to a signaling pathway in a different cell type. We examined IMR90 fibroblasts stimulated for 1 h with tumor necrosis factor alpha (TNFα) [23] and defined a set of TNFα-response genes (see “Methods”). We calculated baseline IMR90 gene PI values from an IMR90 RNAP2 ChIP-seq dataset. Prior to stimulation, TNFα-responsive genes again had a lower distribution of PIs than non-responsive genes (*p* <0.001, Mann–Whitney *U* test; Fig. 3e). TNFα primary response genes, the subset of genes that respond without *de novo* protein synthesis, likewise had a lower PI on average than non-responsive IMR90 genes (*p* <0.001, Mann-Whitney *U* test; Fig. 3f). Together, these analyses suggest that many stimulus-responsive genes are paused but tend to have lower PIs pre-stimulation.

We next asked how changes in RNAP2 pause-release are accompanied by expression changes over time for signal-responsive genes. In VEGFA-stimulated HUVECs, the PI distribution of early upregulated genes shifted leftwards at 1 h and then shifted back towards their basal state at 4 and 12 h (for Hour 1 vs. Hour 0, *p* <0.001, Mann–Whitney *U* test; Fig. 3g). This suggests that VEGFA stimulates a transient increase in RNAP2 pause-release at early upregulated genes, which coincides with the temporal expression profile of these genes. The late upregulated, downregulated, or non-responsive genes did not have a similar shift of PI distribution (Fig. 3h and Additional file 1: Figure S7B, C). We observed a similar PI shift for early-responsive genes in IMR90 cells stimulated by TNFα. The PI distribution of genes upregulated 1 h after TNFα treatment shifted leftward at the same time point (*p* <0.001, Mann–Whitney *U* test; Additional file 1: Figure S7D). These data suggest that signal-induced RNAP2 pause-release participates in rapid stimulus-induced gene upregulation but may be less prevalent among genes with slower signal responses.

To further test the role of increased pause-release for VEGFA-induced gene expression changes, we measured the effect of flavopiridiol (FP), an inhibitor of the P-TEFb pause-release complex, on selected early and late responsive genes. Treatment of HUVECs with FP dramatically suppressed the VEGFA-induced upregulation of early-responsive genes at 1 h (Fig. 3i, see “Methods”), but with much less effect at 4 and 12 h. FP treatment also suppressed the activation of some late-responsive genes at 4 and 12 h (Fig. 3i), although it is unclear whether this effect is solely a consequence of FP treatment or is secondary to disrupted activation of early upregulated genes. Overall, our analyses suggest that RNAP2 pausing selectively regulates rapid activation of transcription in response to stimuli.

#### High RNAP2 TSSR density is associated with increased pause-release

**Fig. 4:**
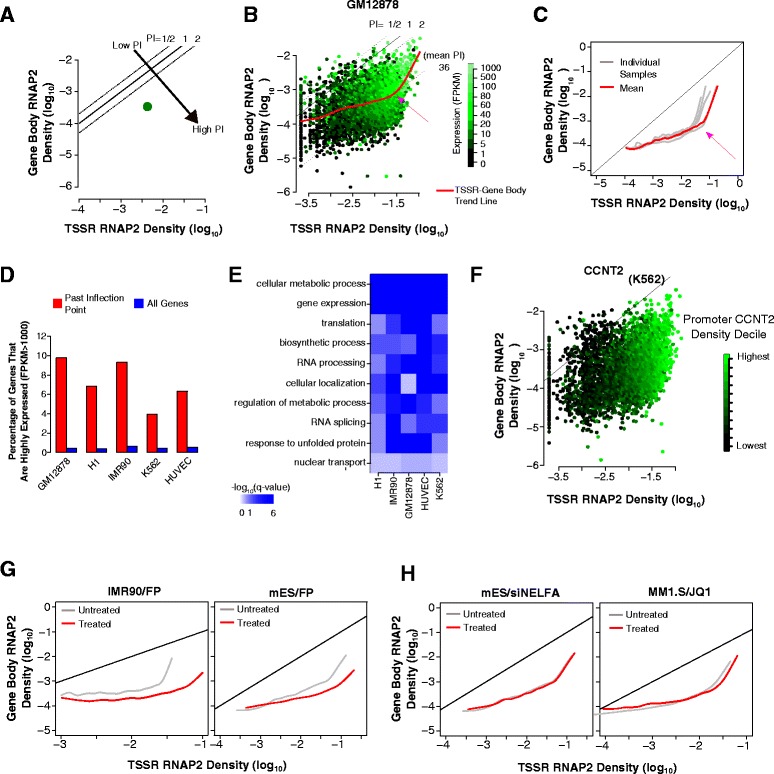
High RNAP2 TSSR density triggers increased RNAP2 gene body density. **a**, **b** Scheme to visualize a gene’s RNAP2 density by its TSSR and gene body density and a third variable, such as gene expression. PI is shown by the gene’s position along indicated diagonals. **a** *Schematic plot* of one gene, colored by its expression level. **b** All genes in GM12878. A smoothed spline *mean trend line* between RNAP2 TSSR and gene body density showed a biphasic slope, with an inflection point (*pink arrow*) and higher slope at high RNAP2 TSSR density. The steeper portion of the trend line suggested that high TSSR RNAP2 density enhanced RNAP2 gene body density. **c** *Mean trend lines* for five different cell types. A *biphasic trend line* with an inflection point at high RNAP2 TSSR density was a common feature of all cell lines. **d** Highly expressed genes (FPKM >1000) were over-represented among genes on the steep portion of the mean trend line, compared to all genes. **e** Functional annotations associated with genes on the steep portion of the mean trend line were consistently enriched for multiple GO biological process terms across several cell types. **f** Promoter density of CCNT2, a pause-release protein, correlated with TSSR RNAP2 density in K562 cells. **g** Inhibition of pause-release abrogated enhanced RNAP2 gene body density at high RNAP2 TSSR density. Treatment with pause-release inhibitor FP flattened the steeper portion of the mean trend line observed in untreated cells in both IMR90 and mES cells. **h** Modulation of pause-release regulators NELFA and BRD4 did not abrogate the inflection point. NELFA was knocked down with siRNA and BRD4 was antagonized with the small molecule JQ1. Neither treatment abolished the inflection point or the steeper portion of the mean trend line

To further explore the relationships among RNAP2 promoter density, RNAP2 gene body density, and gene expression, we plotted the RNAP2 densities at TSSR and gene body region (GBR) of each gene on the two axes (in the log-scale) and colored each point by its expression level (Fig. 4a, b). On this graph, the PI of a gene is constant along a diagonal line since it reflects the ratio between TSS and gene body RNAP2 density (Fig. 4a, b). Taking GM12878 as an example, we observed a wide range of expression levels along a diagonal line, consistent with our previous analysis (Fig. 2b). Next, we sought to determine if increased initiation of RNAP2 (large *x* values) led to increased RNAP2 elongation (large *y* values). After using a cubic smoothing spline to model the trend between TSSR and GBR RNAP2 density, we observed an interesting biphasic relationship (Fig. 4b, red line). Across low and intermediate TSSR RNAP2 densities, the average GBR RNAP2 curve was relatively flat, suggesting that most promoter-proximal RNAP2 that accumulates likely remains paused at the TSS. However, at a high TSSR RNAP2 density, there was a large inflection point in the trend curve (Fig. 4b, arrow), past which the slope of the line increased. This indicates that on average a greater fraction of the promoter-proximal RNAP2 undergoes pause-release and enters the gene body. The same biphasic trend line was observed in the other four cell lines we analyzed (Fig. 4c and Additional file 1: Supplementary Figure S8A), suggesting that this pattern may hold across a range of mammalian cell types. We explored what common functional and expression properties genes past the inflection point have across cell types. As expected, genes to the right of the inflection point were more enriched for highly expressed genes (FPKM >1000; Fig. 4d). Additionally, a number of GO biological process terms were enriched for genes past the inflection point, such as for gene expression, biosynthetic processes, and translation (Fig. 4e).

To understand what proteins might regulate this switch at the inflection point, we investigated some proteins known to regulate RNAP2 pause-release. NELF stimulates RNAP2 pausing, whereas the P-TEFb complex, containing CDK9 and CCNT2 subunits, stimulates RNAP2 pause-release [1]. Examining NELF TSSR occupancy in K562 cells, we find that it is strongly correlated with RNAP2 TSSR density but not with gene body density or PI (Additional file 1: Figure S8B), consistent with prior reports [11, 14]. Repeating this analysis on P-TEFb components CDK9 and CCNT2 revealed that their promoter occupancy is likewise strongly correlated with TSSR RNAP2 density but not to gene body RNAP2 density or PI, with the strongest signal occurring to the right of the inflection point (Fig. 4f and Additional file 1: Figure S8C). Hence, P-TEFb and NELF appear to be recruited to promoters as promoter-proximal RNAP2 accumulates but is not significantly affected by RNAP2 gene body entry or pause-release. These observations suggest that pause-release is regulated at the level of P-TEFb or NELF activity, rather than their recruitment. Furthermore, the promoter occupancy pattern of P-TEFb and NELF suggest that they are appropriately positioned to modulate RNAP2 GBR entry at high TSSR RNAP2 density.

To further investigate the involvement of P-TEFb and NELF in modulating RNAP2 gene body density, we examined available datasets where RNAP2 ChIP-seq was performed in cells treated with NELF siRNA or P-TEFb inhibitors. Treatment with the P-TEFb inhibitor FP in both mouse ES [14] (mES) and human IMR90 fibroblasts [23] flattened the steeper portion of the trend line to the right of the inflection point (Fig. 4g). Since P-TEFb is required for pause-release [1, 2], it is expected that loss of P-TEFb should affect the trend curve as observed. Comparatively, inhibition of BRD4 and ELL3, both of which promote RNAP2 pause-release by modulating P-TEFb activity, or inhibition of NELF did not influence the biphasic trend line (Fig. 4h and Additional file 1: Figure S8D). Increased pause-release at high TSSR RNAP2 density was not likely to result from RNAP2 promoter saturation, since RNAP2 promoter binding could be further increased by FP treatment (Additional file 1: Figure S9). Thus, P-TEFb links high TSSR RNAP2 density to enhanced RNAP2 pause-release, but other components involved in RNAP2 pausing regulation, such as BRD4, NELF, and ELL3, are dispensable for this effect.

Together, our analyses support a non-linear relationship between RNAP density at TSS and gene body, with the increased pause-release triggered at high RNAP2 TSS density mediated by P-TEFb activity.

### Promoter H2A.Z deposition increases with RNAP2 pausing

**Fig. 5:**
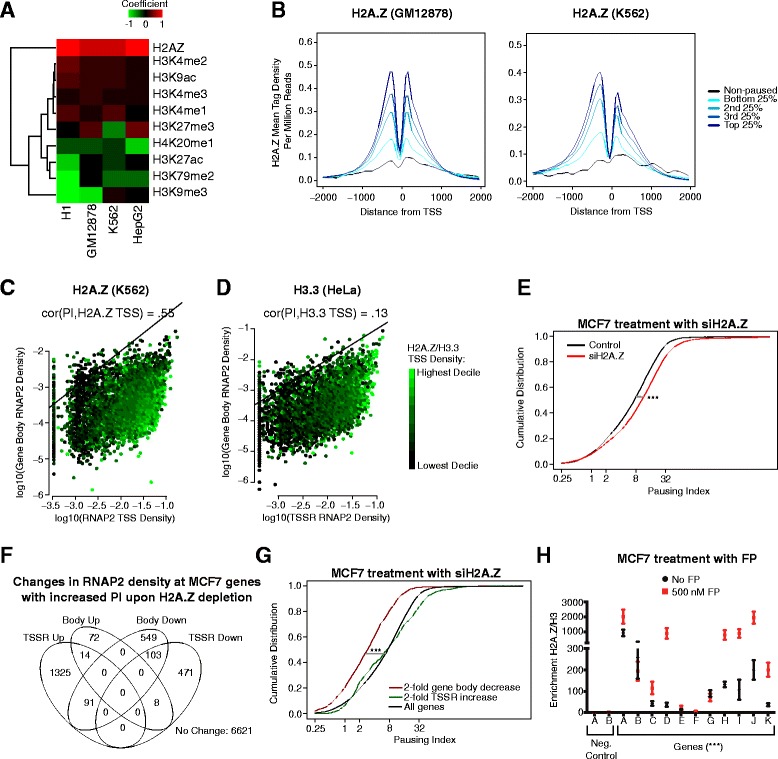
H2A.Z deposition is strongly tied with greater RNAP2 pausing. **a** Correlation of TSS chromatin features with PI in four cell types. Correlation was modeled with a multi-dimensional linear regression. H2A.Z had a strong positive coefficient across all four tested cell types, suggesting that H2A.Z occupancy correlated with increased PI. **b** H2A.Z mean tag density around the TSS in GM12878 and K562 by increasing PI quintile. H2A.Z density increased with increasing PI. **c**, **d** Relationship of H2A.Z or H3.3 TSSR density to RNAP2 TSSR and gene body density. H2A.Z correlated with PI (Spearman r = 0.55) whereas H3.3 did not (Spearman r = 0.13). Genes with the same PI values align on the diagonal; one example line with PI = 1 is shown. **e**–**g** H2A.Z knockdown in MCF7 cells globally increased RNAP2 pausing. MCF7 cells were treated with H2A.Z or control siRNA. RNAP2 pausing was then determined from RNAP2 ChIP-seq. H2A.Z knockdown in MCF7 cells globally increased RNAP2 pausing (**e**; ***p <0.001; Mann–Whitney *U* test). We counted the number of genes with absolute fold-change in RNAP2 density >2 in TSSR or gene body (**f**). Predominantly, genes increased TSSR RNAP2 density or decreased either TSSR or gene body density, but rarely both. Genes with exclusively ≥2-fold decrease in gene body RNAP2 density had a lower PI pre-knockdown than all genes or genes with exclusively ≥2-fold increase in their TSS RNAP2 density, further suggesting that H2A.Z differentially affects genes based on their PI. (**g**; ***p <0.001; Mann–Whitney *U* test.) (**h**). Antagonizing pause-release predominantly increased H2A.Z enrichment at promoters (all 500 NM FP vs. no FP, ***p <0.001; *t*-test). MCF7 cells were treated with FP to block pause-release. H2A.Z occupancy at 11 promoters was measured by ChIP-qPCR and normalized to histone H3 occupancy

As many chromatin remodelers associate with RNAP2 [1], the location of RNAP2 can lead to local chromatin remodeling. Since differences in RNAP2 pausing would change the duration of RNAP2 at the promoter of a gene, we hypothesized that differential rates of RNAP2 pausing may be associated with different chromatin features at a promoter. Consistent with previous reports [9, 11], paused genes (PI >2) had greater nucleosome depletion than non-paused genes (Additional file 1: Figure S10A). Nevertheless, paused genes maintained strong nucleosome signatures near the TSS. Therefore, we further investigated the relationship between chromatin features with PI using a multi-dimensional linear model (Fig. 5a and Additional file 1: Figure S11A). This modeling controlled for potential cofounding effects of correlation between individual chromatin features. The estimated coefficients from the model showed either inconsistent or weak coefficients for each chromatin mark except one: H2A.Z (Fig. 5a and Additional file 1: Figure S11A), a H2A histone variant essential for lineage commitment and embryonic development [24]. This relationship held even when we included the gene expression levels as a variable within the linear model (Additional file 1: Figure S11A). This was surprising because H2A.Z was found to be anti-correlated with RNAP2 pausing in *Drosophila* [12, 25]. Examination of the distribution of H2A.Z around the TSS showed that its focal occupancy at the –1 and +1 nucleosome positions positively correlated with increasing PI (Fig. 5b). H2A.Z deposition more strongly correlated with PI than with TSSR or gene body RNAP2 density (Fig. 5c), unlike what was observed for P-TEFb or NELF TSS occupancy (Additional file 1: Figure S8A, B). H3.3, another histone variant that occupies the TSS of expressed genes [26], poorly correlated with PI (Fig. 5d), suggesting that RNAP2 pausing is associated with incorporation of specific histone variants. Finally, analysis of an existing dataset on the effect of H2A.Z depletion on nucleosome density [24] as a function of PI provided independent support for a positive correlation between H2A.Z occupancy and PI. Since H2A.Z destabilizes nucleosomes, H2A.Z depletion should increase nucleosome density. If H2A.Z occupancy correlates with PI, then this increase of nucleosome density would be expected to be greater at genes with higher PI. Our analysis of nucleosome density in murine ES cells treated with H2A.Z siRNA [24] was consistent with this expectation (Additional file 1: Figure S10B). Taken together, these data show that H2A.Z positively correlated with RNAP2 pausing in mammalian cells.

To further determine whether H2A.Z regulates RNAP2 pausing in mammalian cells, we knocked down H2A.Z with siRNA in MCF7 breast cancer cells and measured RNAP2 occupancy by ChIP-seq (Additional file 1: Figure S11B, C; see “Methods”). This analysis showed that H2A.Z knockdown increased the PI on average (*p* <0.001, Mann–Whitney *U* test; Fig. 5e), suggesting that H2A.Z antagonizes RNAP2 pausing. This result was surprising given the positive correlation between RNAP2 pausing and H2A.Z, but consistent with studies in *Drosophila* that demonstrated H2A.Z antagonizes RNAP2 pausing, likely by destabilizing nucleosomes that impede RNAP2 elongation [12].

To better understand the mechanism by which H2A.Z depletion in MCF7 increased PI, we analyzed the change in RNAP2 density at TSSR and gene body (Fig. 5f). Among genes with greater than twofold change in either category, RNAP2 density either increased in the TSSR or diminished in the gene body, but rarely both (Fig. 5f). Interestingly, the subset that decreased gene body RNAP2 density upon H2A.Z depletion was distinguished by a significantly lower baseline PI compared to all genes or the subset with increased TSSR RNAP2 density (Fig. 5g). These data suggest that H2A.Z likely modulates RNAP2 pausing by at least two mechanisms: one in which H2A.Z inhibits promoter-proximal RNAP2 accumulation and a second in which H2A.Z stimulates RNAP2 entry into the gene body.

We asked how the presence of H2A.Z at TSS antagonized paused RNAP2 (Fig. 5h) yet positively correlated with PI (Fig. 5a–d). We hypothesized that paused RNAP2 accumulation stimulates H2A.Z deposition at promoters, resulting in a negative feedback loop that limits further RNAP2 pausing. To test this hypothesis, we treated MCF7 cells with FP to increase RNAP2 pausing and then measured H2A.Z enrichment at promoters by ChIP-qPCR. Consistent with our hypothesis, we observed that FP treatment increased H2A.Z enrichment in general at the test promoters (all promoters together comparing treated vs. un-treated, *p* <0.001, *t*-test; Fig. 5h). Together our data suggest that H2A.Z destabilizes nucleosomes and antagonizes pausing in mammals, consistent with previous reports [12, 24]. Unlike in *Drosophila*, H2A.Z correlated with RNAP2 pausing in mammals, potentially due to a negative feedback loop in which pausing increases H2A.Z deposition at promoters.

## Discussion

Our approach of analyzing multiple, previously unconnected datasets from diverse mammalian cells and tissues generated novel insights and new directions about the function of RNAP2 pausing. While many studies have elucidated mechanisms of pausing and pause-release generally through biochemical approaches or by focusing on a particular complex involved in the process [1, 3, 6, 10, 11, 27–31], we took a more genomic approach to identify novel functional and regulatory roles for RNAP2 pausing in mammals that would have been difficult to find through previous approaches given their focus. Accordingly, we discovered several previously unidentified patterns in the genome-wide data with respect to gene expression and chromatin structure as well as validated previously observed patterns across many mammalian cells types.

Our analysis suggested that promoter-proximal RNAP2 pausing deployment is consistent across cell types while further highlighting its high prevalence at mammalian genes. Previous studies of a limited number of cell types suggested that many mammalian genes tend to have a paused RNAP2 near their TSS [7, 14, 15]. We confirmed this observation in our analysis across many cell types. Furthermore, the degree of RNAP2 pausing at a gene tended to be consistent across cell types, despite whether the sample was normal or cancerous. This suggested the promoter sequence may play a role in regulating RNAP2 pausing since it would be relatively static across cell types and accordingly we found genes with increased promoter-proximal paused RNAP2 having high GC content and under-representation of TATA motifs. Notably, sequence motifs previously shown to be enriched in paused *Drosophila* promoters, such as the GAGA motif [11, 32, 33], were not enriched in our mammalian data, identifying species-specific adaptations in important promoter sequence motifs. In addition, genes having consistent deployment of RNAP2 pausing across cell types were recurrently enriched for several biological function terms, such as cellular metabolism, DNA repair, protein localization, and cell cycle, while genes with lower levels of RNAP2 pausing across cell types were enriched for biological function terms related to development. Some of these enrichments were recently identified in an analysis of RNAP2 pausing in mouse embryonic stem cells [34], supporting our results. It is unclear how genes involved in the same biological function attain a consistent pattern of stably paused RNAP2, but one possible mechanism could be that genes within the same biological function share promoter sequence motifs to regulate the deployment of stably paused RNAP2. Further experimental dissection of the promoter sequence will reveal whether certain DNA motifs can indirectly modulate RNAP2 pausing at a particular gene.

We studied the relationship between RNAP2 pausing and steady-state and dynamic gene expression across cell types. Consistent with previous studies [14, 16], promoter-proximal RNAP2 pausing was not a strong determinant of a gene’s steady-state expression level. However, it was previously unnoticed that genes with very high and low PIs tended to have a lower expression level, suggesting that a gene having some moderate level of stably paused RNAP2 can better achieve higher expression levels. Despite this, RNAP2 pausing appeared to regulate how a gene was expressed in at least two different ways. First, genes with promoter-proximal RNAP2 pausing tended to have lower cell-to-cell expression variability in a population of cells of the same type. Earlier studies using florescent probes against individual genes in *Drosophila* embryos suggested RNAP2 pausing may dampen expression variability caused by recruiting RNAP2 [2, 17, 27, 29]. Our study provides the first genome-wide support to this observation and within a more homogenous cell population than an embryo. Paused RNAP2 has been proposed to suppress transcriptional noise arising from variability in RNAP2 recruitment to the promoter [2]. Our analysis of the consistency of RNAP2 pausing across cell types, potentially arising from the importance of the promoter sequence, suggests that RNAP2 pausing would likewise be similar across cells in a population, thereby providing a consistent buffer against cell-to-cell transcription variability caused by RNAP2 recruitment. Second, our analyses provided insights on the roles of RNAP2 pausing in stimulus-induced gene expression changes. Paused RNAP2 was broadly deployed at many signal responsive genes, consistent with a recent study in mouse embryonic stem cells [34] and prior work established that RNAP2 pausing participates in stimulus-responsive gene expression change [1, 5, 35]. Our study confirmed this finding and showed that only a subset of stimulus-responsive genes appears to be driven by dramatic increases in pause-release. At the other stimulus-responsive genes with stable paused RNAP2, this pausing likely has an alternative regulatory role, as suggested by our observation that stimulus-responsive genes had a lower PI on average than non-responsive genes under basal (pre-stimulus) conditions. Although further studies are required to determine the functional significance of this finding, one possibility is that the degree of promoter-proximal RNAP2 pausing may modulate a gene’s inducibility by an external signal. Collectively, our studies suggest that RNAP2 pausing modulates gene expression through several potential mechanisms, without being directly correlated to the magnitude of gene expression.

We investigated the relationship between a gene’s RNAP2 density at the TSS and gene body and its occupancy by proteins that regulate RNAP2 pausing and pause-release. Consistent with previous reports, NELF promoter occupancy correlated with RNAP2 TSSR density [11, 14]. Surprisingly, like NELF P-TEFb promoter occupancy also correlated more closely with RNAP2 binding at the promoter than its binding in the gene body or its level of pause-release. This was unexpected because differential P-TEFb recruitment is thought to be a mechanism of differentially regulating RNAP2 pause-release between genes [1]. Our analysis suggests that P-TEFb recruitment to the promoter is better linked with increased RNAP2 initiation, and that differential RNAP2 pause-release may be governed by differences in P-TEFb activity rather than recruitment.

Our analysis revealed a surprising correlation with pause-release and P-TEFb activity: low to moderate RNAP2 TSS densities had a similar average level of RNAP2 in the gene body whereas high RNAP2 TSS density had a dramatic increase in the average RNAP2 entering the gene body. This apparent increase in pause-release at high RNAP2 TSS densities was dependent on P-TEFb, but not NELF or the P-TEFb regulators BRD4 and ELL3. This suggests that pause-release may be triggered by high rates of RNAP2 initiation through unknown mechanisms, leading to very high expression levels. Understanding the molecular mechanism that makes RNAP2 pause-release dependent on RNAP2 promoter density will require further study of P-TEFb recruitment and activation.

Finally, we discovered a positive correlation between a gene’s promoter occupancy by H2A.Z and its PI. This was surprising since previous reports suggested that H2A.Z is anti-correlated with RNAP2 pausing in *Drosophila* [12, 25]. Previously, Rach et al. suggested that, across species, the deposition of H2A.Z at promoters may be more linked to their sequence composition than to their RNAP2 binding [36]. We also observed that promoter sequence composition influences the deployment of promoter-proximal RNAP2 pausing and hence would be anticipated to correlate with H2A.Z deposition. Our analysis does not exclude the possibility that sequence composition effects contribute to the observed correlation between H2A.Z and PI. However, it is unlikely that the positive correlation that we observed is solely due to the influence of promoter sequence composition, as our perturbation experiments argue that RNAP2 pausing directly affects H2A.Z deposition.

Our perturbation experiments yielded paradoxical results: blocking pause-release increased H2A.Z deposition, yet knocking down H2A.Z increased RNAP2 pausing genome-wide, suggesting that H2A.Z antagonizes RNAP2 pausing, as observed in *Drosophila* [12, 25]. One model that reconciles these data is that there is a negative feedback loop between RNAP2 pausing and H2A.Z deposition where RNAP2 pausing stimulates H2A.Z deposition that in turn hinders further RNAP2 pausing deployment. Such a negative feedback loop may help to fine-tune the extent of promoter-proximal RNAP2 pausing at a gene. Notably, H2A.Z has been previously implicated in aiding transcription by destabilizing nucleosomes and recruiting complexes that facilitate pause-release [12, 24, 37], supporting a negative feedback loop between these two factors. Additional studies will be needed to further test this hypothesis and to elucidate its molecular underpinnings.

## Conclusions

Meta-analysis of 85 human and mouse RNA polymerase II ChIP-seq datasets have revealed insights into the impact of polymerase II pausing on gene regulation.  These include prevalence of pausing, its association with specific biological processes, contributions to reduced expression variability among individual cells in a population, nonlinear impact on expression level, relationship to rapid gene activation, and correlation with promoter H2A.Z occupancy.

## Methods

### Data collection and processing

#### Experimental data generation

##### Cell treatment

MCF7 cells were cultured in DMEM plus 10 % fetal bovine serum. For siRNA knockdown experiments, 10 μM scramble or H2A.Z siRNA (5′-CAGGACUCUAAAUACUCUATT-3′) (Qiagen) were transfected with silentFect™ lipid (Biorad). The cells were collected for western blotting and ChIP 48 h after transfection.

For pause-release inhibition experiments, MCF7 cells were treated with 500 nM flavopiridol (Sigma) or vehicle (DMSO) for 2 h and then collected for H2A.Z and histone H3 ChIP-qPCR.

##### H2A.Z extraction and western blot

In total, 2 × 10^7^ MCF7 cells transfected with control or H2A.Z siRNA were washed once with cold PBS and then lysed with PBS containing 0.5 % TritonX-100, 2 mM PMSF, 5 mM sodium butyrate, and protease inhibitors. To extract histones, the pellet was further incubated with 0.4 M H_2_SO_4_ for 2 h at 4 °C.

The extracted protein was separated on a 4–15 % SDS-PAGE gel, transferred to a PDVF membrane, and probed with H2A.Z antibody (ab4174, Abcam) or Histone H3 antibody (ab1791, Abcam) to detect protein expression.

##### Chromatin immunoprecipitation followed by high-throughput sequencing (ChIP-seq)

The ChIP assay was executed as described previously [21]. In brief, 50 million MCF7 cells treated with either H2A.Z siRNA or FP were crosslinked with 1 % formaldehyde for 10 min at room temperature. Nuclei were extracted with nuclear extraction buffer (20 mM HEPES-KOH pH 7.5, 10 mM KCl, 1 mM EDTA, 0.2 % NP40, 1 mM DTT, and 10 % glycerol) and resuspended in 1 mL sonication buffer (20 mM Tris-HCl pH 8, 150 mM NaCl, 2 mM EDTA, 0.1 % SDS, 1 % TritonX-100). The chromatin was sheared in a Misonix Sonicator 3000 and then incubated with H2A.Z antibody (ab4174, Abcam) or an antibody against RNAP2 C-terminal domain (05-623, Millipore) for overnight immunoprecipitation with protein G Dynabeads (Life Technologies). Beads were then washed with RIPA buffer (50 mM HEPES-KOH pH 7.5, 500 mM LiCl, 1 mM EDTA, 1 % NP40, and 0.7 % Na-Deoxycholate).

ChIP-seq library construction was performed with NEBNext® ChIP-Seq Library Prep Reagent Set (E6200, New England Biolab) following the protocol in the kit with some modifications. Size selection after adaptor ligation was carried out by 2 % agarose gel electrophoresis rather by dual clean up with AMPure XP beads. Libraries were single-indexed PCR primers as we described previously [21]. The final pooled indexed libraries were sequenced on a HiSeq2500 (Illumina; 50 nt single end sequencing). The data are available from GEO GSE60872 or from the Cardiovascular Development Consortium server (https://b2b.hci.utah.edu/gnomex/; sign in as guest).

##### ChIP-qPCR

As described previously [21], immunoprecipated DNA and input DNA were amplified with SYBR master mix (Life Technologies) and quantified on a CFX96 Real-Time system (Bio-Rad). ChIP enrichment was calculated by normalization with input and unbound control regions. Primers used for detection of H2A.Z signal are documented in Additional file 4: Table S3.

#### Obtaining publicly available data

We downloaded and collected data from a variety of sources, include ENCODE (human and mouse). All datasets used in the analysis and their related GEO accession numbers are listed in Additional file 2: Table S1. In general, ChIP-seq datasets were downloaded in FASTQ format (or SRA format then converted to FASTQ). RNA-seq samples from ENCODE were downloaded in their pre-aligned state as BAM files.

In addition to the list of datasets used, a summary of all of the cell types analyzed in this study and related abbreviations are included in Additional file 2: Table S1 in a separate spreadsheet tab.

#### Alignment and processing of RNAP2 and other ChIP-seq datasets

We aligned each human and mouse ChIP-seq or input FASTQ file to hg19 and mm9, respectively, using Bowtie2 [38] with the following parameters: -p 8 –M 1. We processed the aligned reads and filtered low quality mapped reads using the R package *spp.* [39] We calculated the binding characteristics of each factor using *get.binding.characteristics* setting srange = c(50,500) and bin = 5. We filtered low-quality reads passing the aligned reads through *select.informative.reads* and *remove.local.tag.anomalies*. We collected the remaining reads and used them for all further analyses.

#### Alignment and processing of RNA-seq data files

For the human RNA-seq samples from ENCODE provided by Cold Spring Harbor Labs, the alignments were performed with STAR against hg19 and can be downloaded here (http://it-collab01.cshl.edu/shares/gingeraslab/www-data/dobin/ENCODE2/Public/). Other single- or paired-end RNA-seq datasets were aligned with Tophat2 [40] against hg19 or mm9 and the RefSeq transcriptome reference. Tophat2 was run with standard options including –G (for the RefSeq transcriptome).

After alignment, gene FPKM values were quantified in both human and mouse using Cufflinks2 [41] via the *cuffdiff* command with the appropriate RefSeq transcriptome reference file. When a sample had replicates, they were entered jointly into *cuffdiff* to estimate the FPKM for the sample as a whole. Standard options were used otherwise.

### Estimating promoter-proximal RNAP2 pausing

#### Calculation of PI from RNAP2 ChIP-seq data

To calculate pausing index, we adapted the procedure in Lin et al. [13] because we found that it worked best to capture the relevant RNAP2 ChIP-seq signal throughout our datasets. To determine the background-subtracted RNAP2 ChIP-seq read densities, for each annotated RefSeq isoform we mapped the aligned and filtered RNAP2 ChIP-seq and input reads to the TSSR (–50 bp to +300 bp around TSS) and the gene body (+300 downstream of the TSS to +3 kb past the TES). See Fig. 1a and Additional file 1: Figure S1a for a graphical reference. We removed all genes that overlapped each other, were within 3 kb of another annotated gene, or were less than 1 kb in length. To remove potential PCR duplicates, we collapsed reads mapped to the same position into one. After mapping the reads to each region, we normalized the read density by the length of the region and by the number of mapped filtered reads for that particular ChIP-seq or input library multiplied by 1 million (rpm/bp). Then we subtracted the normalized ChIP signal in each region against the normalized input signal in each region. Using background-subtracted RNAP2 ChIP-seq read densities for the TSSR and gene body of each annotated RefSeq, we calculated pausing index (PI) as follows:

$$ Pausing\  Index\ (PI)=\frac{ReadCount(TSSR)/L1}{ReadCount\left( Gene\  Body\right)/L2} $$

Where L1 is the length of the gene TSSR region (always 350 bp) and L2 is the length of gene body region (+300 bp past TSS to 3 kb past the TES of the gene).

To consolidate PI values from multiple RefSeq isoforms for the same gene, we selected the “optimal” isoform for each gene by identifying which annotated RefSeq TSS had the strongest RNAP2 ChIP-seq signal in the TSSR, provided it had at least 0.001 rpm/bp in the TSSR. If no TSSR met this criterion, we searched for the optimal TSS by finding a TSSR with a minimum fourfold H3K4me3 enrichment (normalized ChIP/input) at the promoter (+/– 500 bp around each annotated TSS). We chose the fourfold threshold because H3K4me3 enrichment at TSSs across the genome for multiple cell lines was bi-modally distributed. The fourfold enrichment threshold divided the two different modes fairly evenly and consistently across samples. If a gene had no annotated TSS that passed either of these criteria, we did not assign it a PI value (included as part of “non-paused”). If multiple RefSeq isoforms used the same annotated TSS, we selected the longest annotated isoform in order to capture all available RNAP2 signal over the span of the gene.

#### Reproducibility across different laboratories/antibodies

We analyzed the reproducibility in calculating RNAP2 density and subsequently the PI across replicates, especially across different antibodies. We found that within our broad TSSR and gene body regions that the PI tended to strongly correlate and have a monotonic increasing relationship between replicates and across different antibodies (see Additional file 1: Figure S2). As such, we then ran most analyses on one of replicates performed with the 8WG16 RNAP2 antibody (as long as one was available).

#### Defining pausing and pausing gene sets

We used a minimum PI threshold of 2 to define paused genes across datasets. There have been multiple ways of defining the paused threshold with similar values [8, 13–15]. With our filtering procedure to remove low signal genes, we have sufficient read density to have a high confidence estimate of this ratio in most cases. Genes with either PI less than 2 or no assigned PI were considered non-paused.

To determine strongly and weakly paused genes, we grouped all isoforms across different cell types into a single matrix with the PI from the corresponding cell type (NA if that isoform is not used in that sample). Within a particular sample, genes were ranked by their PI, with NAs taking the lowest rank. Then we calculated the median PI rank for each isoform across genes. We assembled the list of all paused genes by identifying all genes that were paused in at least one sample. Then highly and lowly paused genes were generated from taking the upper and lower half of the ranked median PIs, as long as they were also part of the all paused genes set.

#### Plotting each gene on a 2D graph (TSSR-gene body RNAP2 density plot)

To visualize each gene by its TSSR and gene body RNAP2 density while also viewing its PI and a third variable, e.g. gene expression, we plotted each gene with an assigned PI on a 2D graph by its TSSR and gene body RNAP2 density. To reduce over-plotting of data points, we binned nearby genes using the R package *hexbin*, which groups genes into 2D bins, and plotted the location of each bin on the graph. When coloring each point by a third variable, we calculated the mean of the third variable for genes within the same bin and colored the plotted point accordingly.

### Additional analyses

#### Multiple testing correction procedure

For panels where there are more than one statistical tests being performed, we verified that all tests passed at least the family-wise error rate (FWER) threshold by estimating the Bonferroni threshold for that number of hypotheses/tests performed (where the alpha was 0.05). If the *p* value of any test did not pass the FWER threshold, then we did not report the *p* value as significant. Otherwise, a significant *p* value reported is the value from the statistical test. For panels where the number of hypotheses tested was greater than 10 (e.g. GO analysis), we controlled for the false discovery rate by converting *p* values into q values as described in the next section.

#### Gene Ontology (GO) enrichment analysis and cancer gene overlap

Biological process GO enrichment analysis was performed using the *topGO* Bioconductor package (http://www.bioconductor.org/packages/release/bioc/html/topGO.html). GO annotations were based on the *org.Hs.eg.db* Bioconductor packages for human. All tests were performed using all RefSeq genes. *P* values were calculated from the set of enriched GO terms using Fisher’s exact test. For each sample, *p* values were converted into q values using the *qvalue* Bioconductor package using default parameters over the range of all tested GO terms.

#### Calculating sequence composition enrichment

We analyzed the sequence content around the promoter (±500 bp around TSS) using the UCSC hg19 or mm9 sequence assembly, as appropriate. For genes with a PI, we used their assigned primary RefSeq annotated TSS. For remaining genes, we assigned each the first annotated RefSeq TSSs. For the GC and CpG ratio analyses, we first corrected the RNAP2 TSSR density for genes with an assigned PI by the GC content within the TSSR (–50 bp to +300 bp around TSS) from the appropriate assembly. We corrected the TSSR RNAP2 density by fitting a smooth spline from the TSSR percent GC content to the log10 TSSR RNAP2 density. We then generated the predicted log_10_ TSSR RNAP2 density from the fitted model and subtracted it from the measured log_10_ TSSR RNAP2 density such that GC content and TSSR RNAP2 density no longer correlated. We then recalculated the PI for genes with an assigned PI using the corrected TSSR RNAP2 density as otherwise described in Section 2 of the Supplement and also recalculated the sets of strongly and weakly paused genes across all human and mouse samples with the updated PIs. Using these corrected PIs and gene set for this analysis, we then calculated the percent GC content and CpG ratio of each promoter region. The CpG ratio was calculated as:

$$ \begin{array}{c}\hfill N= Promoter\  Length=1000\  bp\hfill \\ {}\hfill CpG\  Ratio = \frac{\raisebox{1ex}{$ Number\  of\ CG\  and\ GC\  dinucleotides$}\!\left/ \!\raisebox{-1ex}{$N$}\right.}{\raisebox{1ex}{$ Number\  of\ Cs$}\!\left/ \!\raisebox{-1ex}{$N$}\right.*\raisebox{1ex}{$ Number\  of\ Gs$}\!\left/ \!\raisebox{-1ex}{$N$}\right.}\hfill \end{array} $$

After calculating and gathering the distribution of percent GC content and CpG ratio values for the different gene categories, they were compared using the Mann–Whitney *U* test.

For the sequence enrichment analysis, all paused genes were used in this analysis in their original, uncorrected form (as correcting for GC content in the TSSR region had minor effect on the result, data not shown). We generated all 5- and 6-mers for the standard DNA bases. For each n-mer, we counted its occurrence in the promoter region in paused remaining genes. Then we compared the distribution of counts using the Mann–Whitney *U* test. To estimate an enrichment score to order the over-representation of n-mers in paused or non-paused gene sets, we used the –log10 *p* value of the Mann–Whitney *U* test. If the distribution of counts was greater for the paused genes, we gave the *p* value a positive sign. Otherwise, we gave the *p* value a negative sign. (A positive sign if no difference was detected.) Each n-mer was plotted in order by their enrichment score, with percent GC content and the presence of a CpG and/or TATA in the n-mer (both binary values).

#### Paused versus non-paused expressed genes

For each analyzed cell type, we collected genes with cufflinks-processed FPKM value greater than or equal to 1. Among these genes, those with PI >2 were defined as paused and the rest were non-paused. For the single-cell variability analysis, we used the separate single-cell RNA-seq data with their corresponding cufflinks-processed FPKM values for each cell in both replicates of GM12878 [18] and H1 [19]. Expressed genes were those with mean FPKM across individual cells of at least 1. Paused and non-paused states were assigned based on GM12878 and H1 RNAP2 ChIP-seq data, as described above. We divided expressed genes into 20 % quantiles (using R’s *quantile* function with default parameters) by their mean FPKM value. For paused and non-paused genes in each quantile, we calculated the coefficient of variation (SD/mean) of each gene across all individual cells in a population and tested whether the coefficient of variations distributions were different between paused and non-paused genes by the Mann–Whitney *U* test.

#### PI-expression trend modeling

For the RNAP2 TSSR, gene body, total density, or PI to gene expression analyses, we used Spearman correlation given the non-linear distribution of data points. Individual trend lines were calculated using a smoothed cubic spline (via *smooth.spline* function in R) with no preset degrees of freedom. Quantile trend lines were generated by calculating a running 25 % and 75 % quantile (using *runquantile* from the R package *catools*) followed by smoothing. To accommodate regions of low sample density, the maximum or minimum values were set to be no higher or lower than the mean trend line.

The aggregate plot of multiple cell types was also created using cubic splines but using a low degree of freedom (df = 4) to emphasize the hill-shaped pattern that described the vast majority of the trend rather than the variability in the extremes.

#### Inflection point analysis

We estimated the trend curve between TSSR and gene body RNAP2 density by modeling the smooth spline between the two variables using *smooth.spline* with default parameters. We estimated the trend line on the log10 scale, to highlight the rapid uptick at the high TSSR RNAP2 density. We could visualize the inflection point on the TSSR-gene body RNAP2 plot, and estimate the TSSR RNAP2 density by calculating the maximum second derivative value using the *predict* function.

#### Stimulus responsive analysis

Using cufflinks-generated FPKM quantification of gene expression levels, we calculated responsive gene sets based on FPKM fold-change relative to baseline (e.g. Hour 0). We filtered out any genes that did not have a FPKM of at least 1 in any of the time points available for a dataset. We divided the responsive genes into the following groups: early, minimum twofold increase in FPKM within 1 h post stimulation; late, minimum twofold increase in FPKM in the later time points and not within the first hour; downregulated (down), minimum twofold decrease in FPKM in any time point; or non-responsive, expressed throughout the time course but never any twofold expression change. The “responsive” gene category was the aggregate of early, late, and downregulated genes for a particular time series dataset.

To test for significant differences between responsive and non-responsive genes at baseline, we compared the PI distribution of genes in each category in the unstimulated state using the Mann–Whitney *U* test. To test for significant changes in the PI distribution over time, we compared each non-baseline time point to baseline using the Mann–Whitney *U* test. There were many more non-responsive genes than responsive genes, so for statistical testing we subsampled a number of genes equivalent to the total number of responsive genes from each time point in the dataset.

#### Chromatin structure analysis

To calculate enrichments for chromatin features, we calculated the fold-enrichment for ChIP reads over input reads, with each normalized to their respective number of mapped reads. For analysis of correlation between each chromatin mark and gene PI, we calculated input-subtracted ChIP read density at each promoter (±500 bp around the TSS) that matched the optimal RefSeq used for the PI calculation (see “Calculation of PI from RNAP2 ChIP-seq data” above). We estimated the chromatin enrichment, positive or negative, for PI by training a multi-dimensional linear model on each of the log2 estimated enrichment ratios for each chromatin mark studied (e.g. H2A.Z) to predict the log2 PI value for each gene. Each linear model was trained on each cell type displayed in Fig. 5a individually. The coefficients of the linear model were used to plot the matrix in Fig. 5a. In Additional file 1: Figure S11A, we performed the same analysis, but included the gene expression level (measured from RNA-seq data in FPKM on the log10 scale) as another variable within the model.

To calculate the average density profiles, we binned genes into 25 % quantiles either by their PI or gene expression level, as indicated by the particular graph. We mapped reads for genes within each quantile to 50 bp bins (or 10 bp bins for the MNase nucleosome density analysis, since it was more deeply sequenced) around the TSS and estimated the mean read density per bin normalized to the library size times 1,000,000 reads (reads per million). All profiles were smoothed in a similar way, by passing span = 0.1 to the *loess* function in R.

### Data access

New high throughput data in this manuscript are available at Gene Expression Omnibus (GEO) under accession code GSE60872 or on the Cardiovascular Development Consortium server at: https://b2b.hci.utah.edu/gnomex/. The accession codes for new and previously published high throughput data are listed by sample in Additional file 2: Table S1.

## Abbreviations

EBV, Epstein Barr virus; FP, flavopiridol; GO, Gene Ontology; NELF, negative elongation factor; PI, pausing index; P-TEFb, positive transcription elongation factor b; RNAP2, RNA polymerase II; TAD, topologically associating domain; TES, transcription end site; TNFα, tumor necrosis factor α; TSS, transcription start site; VEGFA, vascular endothelial growth factor A.

## Additional files

Additional file 1: Figure S1. Measurement of pausing index (PI). **Figure S2.** Robustness of RNAP2 pausing calculations. **Figure S3.** Pausing across cell and tissue types. **Figure S4.** Correlation between pausing and promoter GC and CpG content **Figure S5.** Relationship between whole gene, TSSR, and gene body RNAP2 density and PI to gene expression for GM12878, H1, K562, IMR90, HUVEC, and HepG2 cells. **Figure S6.** Grouping paused and non-paused genes by mean population-wide expression shows no consistent expression level difference within quantile. **Figure S7.** Effect of extracellular stimuli on RNAP2 pausing. **Figure S8.** Relationship of gene expression to TSSR and gene body RNAP2 density and to PI. **Figure S9.** The inflection point does not appear to be driven by a limit to the level of initiating RNAP2. **Figure S10.** Nucleosome positioning and RNAP2 pausing. **Figure S11.** Chromatin features and RNAP2 pausing. (PDF 7287 kb) Additional file 2: Table S1. Datasets used in this study. (XLSX 19 kb) Additional file 3: Table S2. GO biological process enrichment analysis for the top and bottom 25 % of genes by PI for each cell type. (XLSX 23028 kb) Additional file 4: Table S3. List of primers used in this study. (XLSX 10 kb)
