# Supplementary material for: Comprehensive analysis of promoter-proximal RNA polymerase II pausing across mammalian cell types
Source: Genome Biol. 2016 Jun 3;17:120. doi: 10.1186/s13059-016-0984-2 (PMC4893286; doi:10.1186/s13059-016-0984-2)
Supplement: Additional file 1: Figure S1. — Measurement of pausing index (PI). Figure S2. Robustness of RNAP2 pausing calculations. Figure S3. Pausing across cell and tissue types. Figure S4. Correlation between pausing and promoter GC and CpG content Figure S5. Relationship between whole gene, TSSR, and gene body RNAP2 density and PI to gene expression for GM12878, H1, K562, IMR90, HUVEC, and HepG2 cells. Figure S6. Grouping paused and non-paused genes by mean population-wide expression shows no consistent expression level difference within quantile. Figure S7. Effect of extracellular stimuli on RNAP2 pausing. Figure S8. Relationship of gene expression to TSSR and gene body RNAP2 density and to PI. Figure S9. The inflection point does not appear to be driven by a limit to the level of initiating RNAP2. Figure S10. Nucleosome positioning and RNAP2 pausing. Figure S11. Chromatin features and RNAP2 pausing. (PDF 7287 kb) [file 13059_2016_984_MOESM1_ESM.pdf]

Supplementary Information

Day et al., Comprehensive Analysis of Promoter Proximal RNA Polymerase II Pausing Across Multiple Mammalian Cell Types

Contents

Supplementary Tables (please see separate excel spreadsheets)

Supplementary Table 1 – Datasets used in this study.
Supplementary Table 2 – GO biological process enrichment analysis for the top and bottom 25% of genes by PI for each cell type.
Supplementary Table 3 – List of primers used in this study.

Supplementary Figures

Supplementary Fig. 1 – Measurement of pausing index (PI).
Supplementary Fig. 2 – Robustness of RNAP2 pausing calculations.
Supplementary Fig. 3 – Pausing across cell and tissue types.
Supplementary Fig. 4 – Correlation between pausing and promoter GC and CpG content
Supplementary Fig. 5 – Relationship between whole gene, TSSR, and gene body RNAP2 density and PI to gene expression for GM12878, H1, K562, IMR90, HUVEC, and HepG2 cells.
Supplementary Fig. 6 – Grouping paused and non-paused genes by mean population-wide expression shows no consistent expression level difference within quantile.
Supplementary Fig. 7– Effect of extracellular stimuli on RNAP2 pausing.
Supplementary Fig. 8–Relationship of gene expression to TSSR and gene body RNAP2 density and to PI.
Supplementary Fig. 9– The inflection point does not appear to be driven by a limit to the level of initiating RNAP2.
Supplementary Fig. 10 – Nucleosome positioning and RNAP2 pausing.
Supplementary Fig. 11 – Chromatin features and RNAP2 pausing.

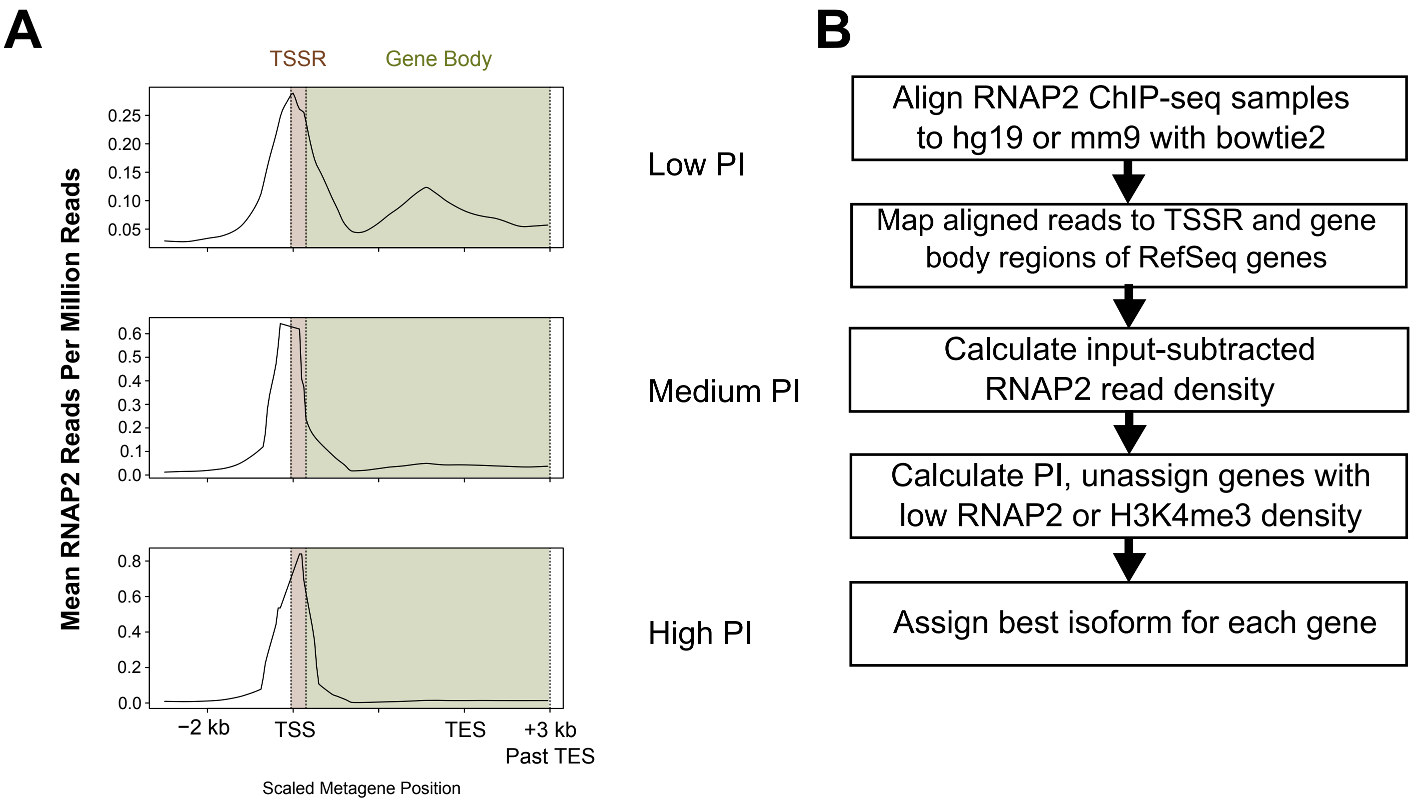

Supplementary Fig. 1. Measurement of Pausing Index (PI). A. Smoothed metagene profiles showing RNAP2 averages of genes divided into the top, middle, and bottom terciles by PI in GM12878. TSSR, the transcriptional start site (TSS) region, was defined as TSS -50 to +300 bp. Gene body was defined as TSS+300 bp to transcriptional end site (TES) + 3 kb. **B.** Workflow for processing RNAP2 ChIP-seq data and assigning a PI value to each gene in the genome with sufficient RNAP2 or H3K4me3 density at the TSS.

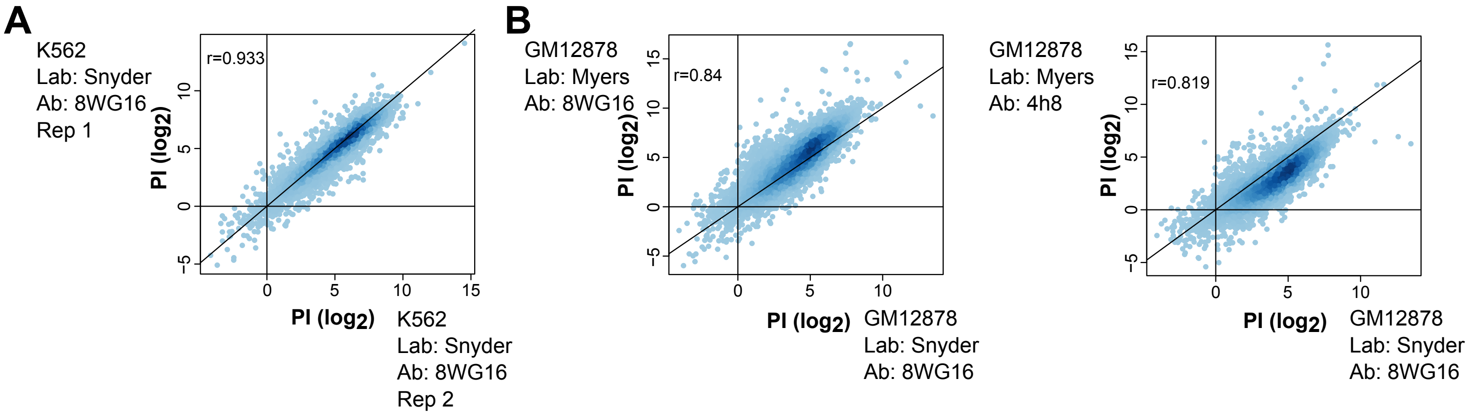

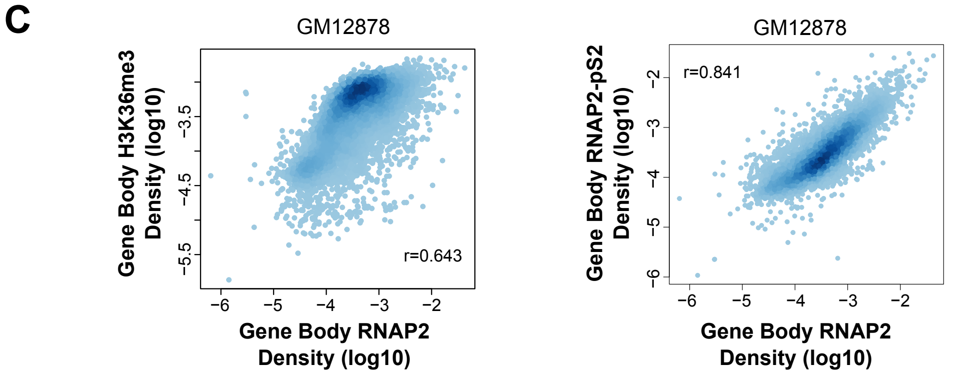

D

	RNAP2 vs.	
	H3K36me3	RNAP2 pS2
GM12878	0.643	0.841
K562	0.650	0.831
HepG2	0.610	0.840

Supplementary Fig. 2. Robustness of RNAP2 pausing measurements and calculations. A-B. Comparison of RNAP2 PI calling across biological replicates from the same and different labs and across different RNAP2 antibodies. A, Biological replicates of the K562 cell line from the same lab with the same antibody. B, Biological replicates of the GM12878 cell line from different labs using the same antibody, or from different labs using different antibodies. **C-D.** Gene body RNAP2 density correlation with H3K36me3 and RNAP2 pS2 across samples. Pearson correlations are shown throughout this figure.

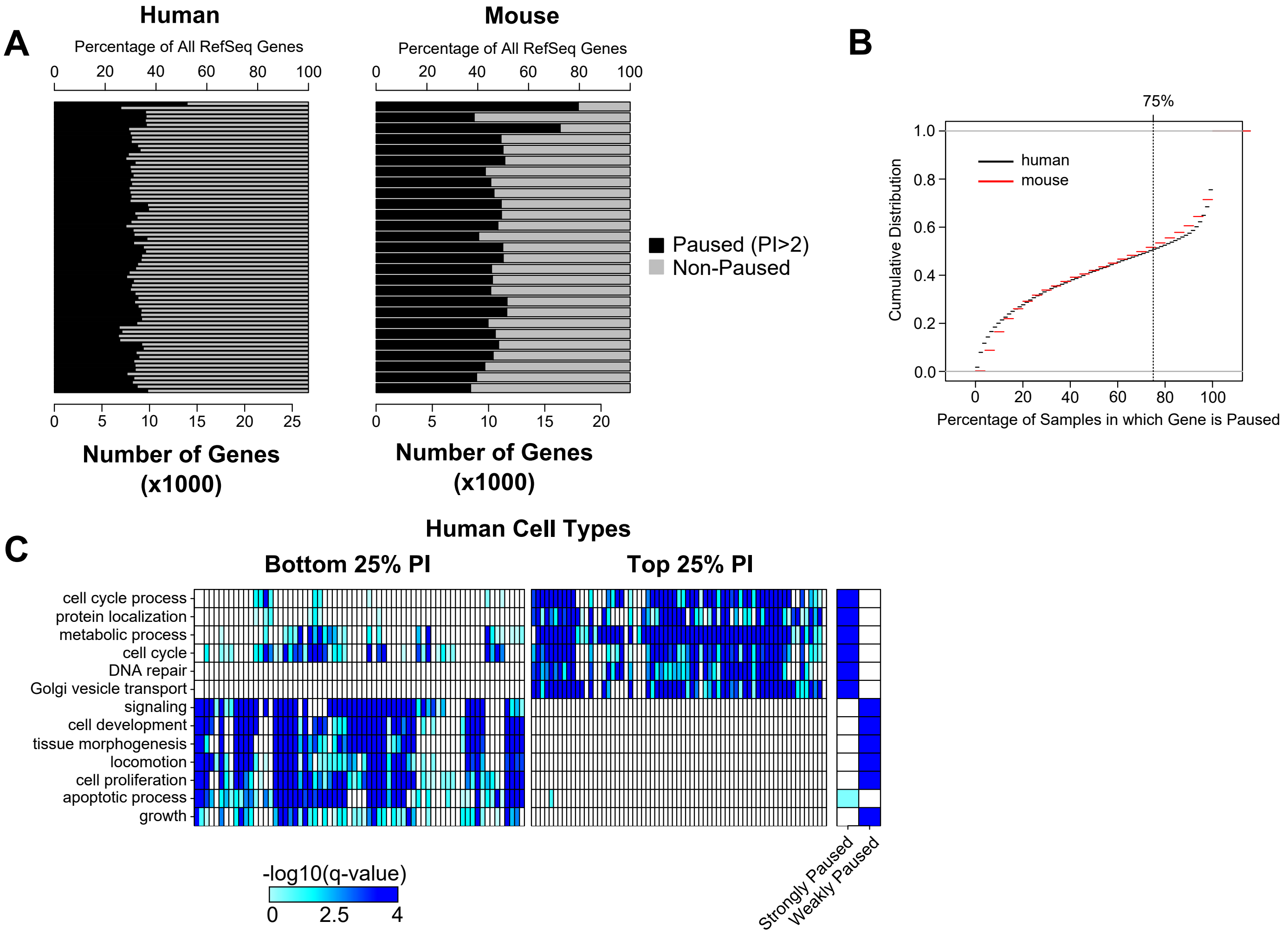

Supplementary Fig. 3. Pausing across cell and tissue types. A. Extended distribution of paused and non-paused genes across all analyzed RNAP2 ChIP-seq samples (expands Fig. 1b, first two rows of each set are "Total" and "Shared"). **B.** Empirical cumulative distribution of the percentage of samples analyzed in which a gene is paused. The majority of paused genes are paused in 75% of samples or mor. **C.** Extended GO term analysis of the genes with the top and bottom quartile of PI in individual human samples, and of genes strongly or weakly paused across all human samples (see Methods).

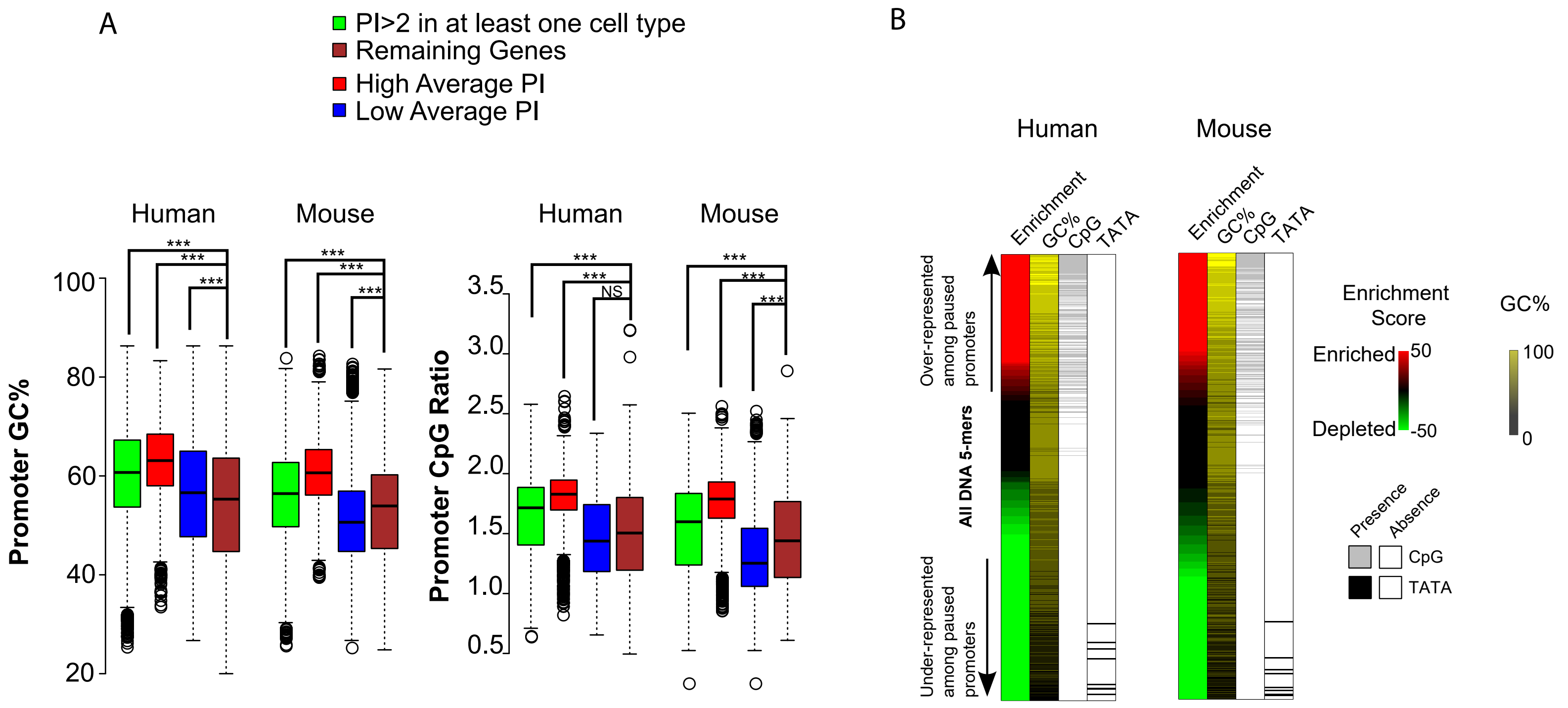

Supplementary Figure 4. Correlation between RNAP2 Pausing and Promoter GC and CpG Content.

A. The promoter content of genes with a PI>2 in at least one cell type tended to have a significantly higher GC and CpG content than the remaining set of genes. This effect was more specific to genes with a higher average PI across cell types, however, than genes with a lower average PI across cell types. **B.** Analysis of promoter composition using 5-mers, analogous to the analyses of Fig. 1F using all 6-mers. The 5-mer analysis reproduced the major findings that paused promoters are over-represented for high GC and CpG content and under-represented for the TATA motif.

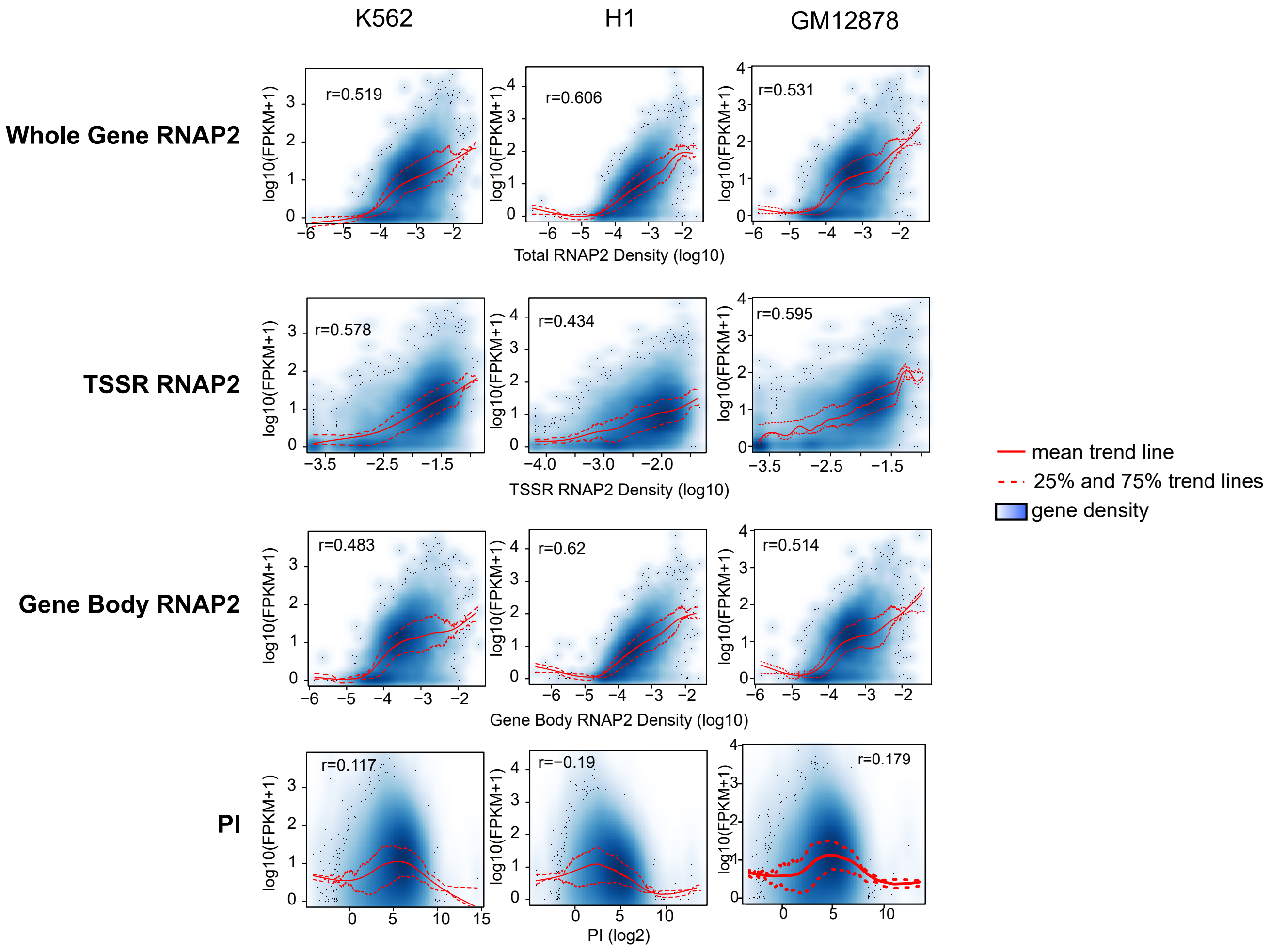

Supplementary Fig. 5 (page 1 of 2). Relationship between whole gene, TSSR, and gene body RNAP2 density and PI to gene expression for H1, K562, IMR90, HUVEC, and HEPG2 human cells. We calculated the trend between each indicated parameter and gene expression levels (similar to Figure 2B).

Overall, we consistently observed that RNAP2 density best correlates with gene expression levels whereas the PI correlated less well. Furthermore, we observed a consistent "hill-shaped" relationship between gene expression and PI, suggesting that within a cell type genes with relatively high and low PI values tended to be not as strongly expressed as genes with intermediate PIs.

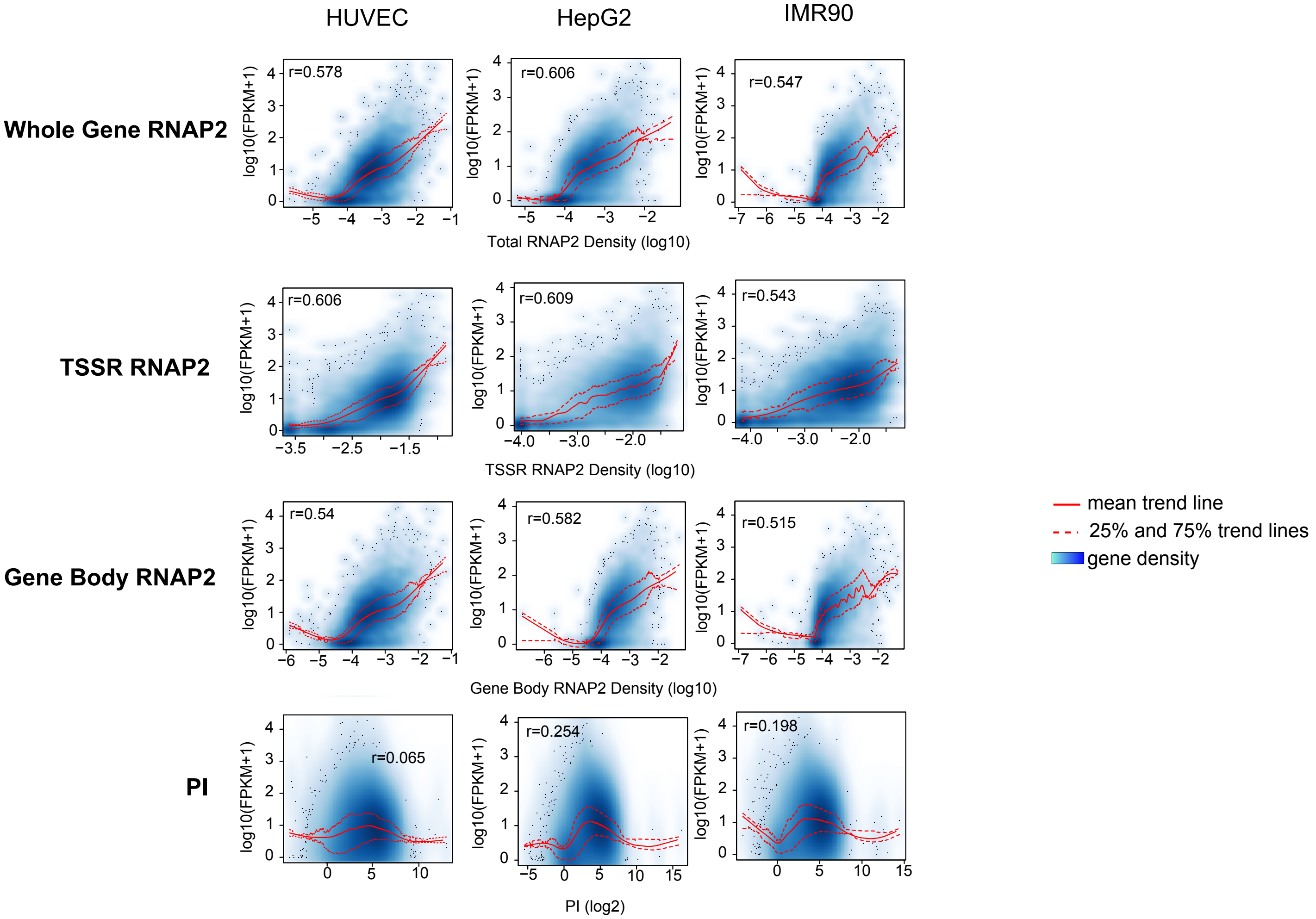

Supplementary Fig. 5, continued (page 2 of 2).

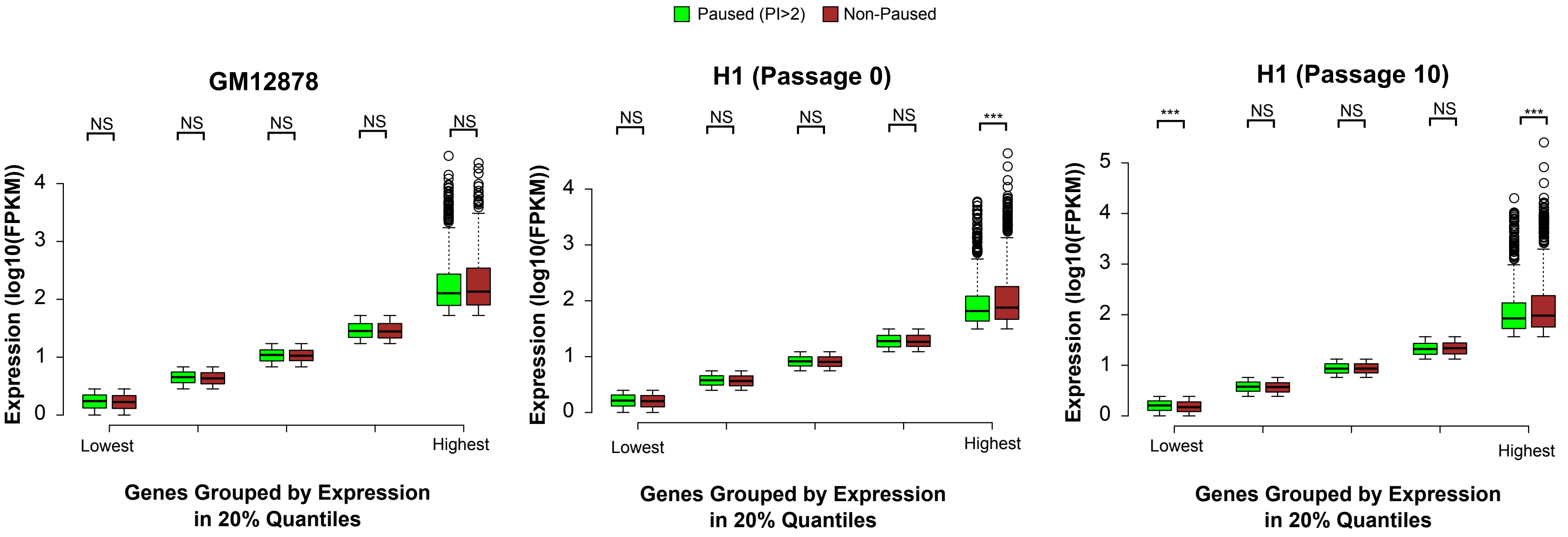

Supplementary Figure 6. Grouping paused and non-paused genes by mean population-wide expression shows no consistent expression level difference within quantile. While comparing the expression coefficient of variation between paused and non-paused genes showed a significant difference (Figure 2C), differences in expression level between paused and non-paused genes within the same 20% quantile did not systemically contribute to this difference since almost all quantiles lacked significant differences in mean population-wide expression level when compared to the Bonferroni multiple testing threshold .01 (Mann-Whitney U test, *** p < .001, NS = not significant).

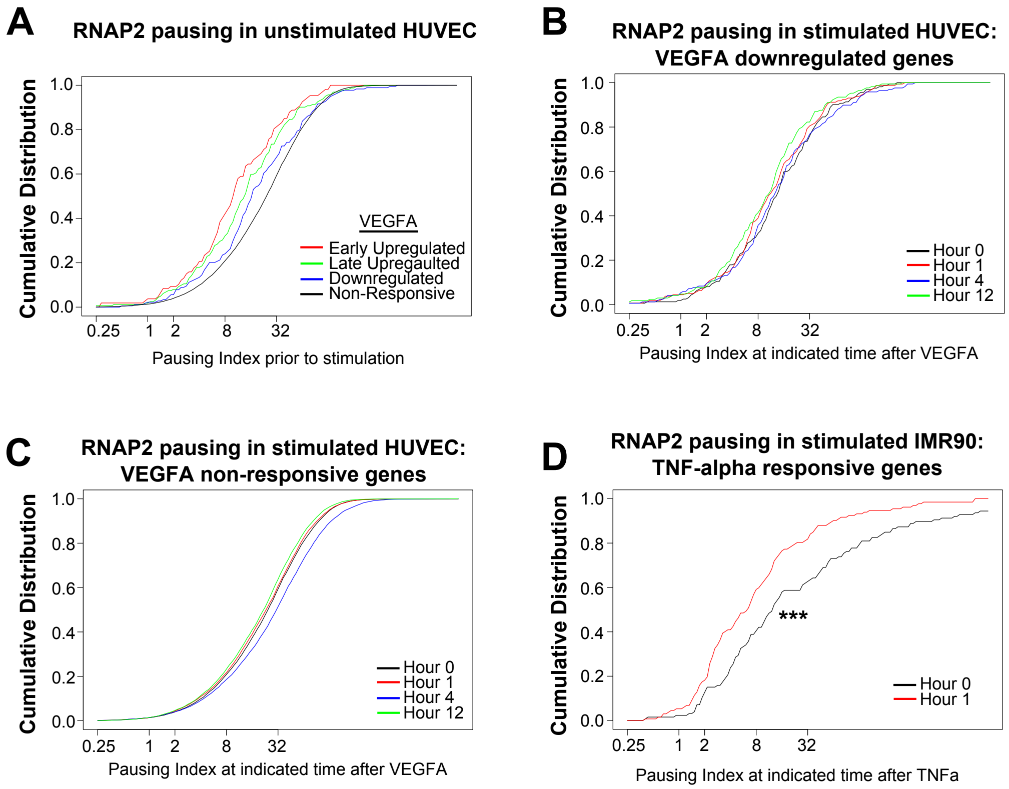

Supplementary Figure 7. Effect of extracellular stimuli on RNAP2 pausing. A. Each of the three categories of VEGFA-responsive genes were less paused than the non-responsive genes in HUVECs in their basal (unstimulated) state. **B-C.** The PI distribution of down-regulated and non-responsive genes did not change substantially during VEGFA stimulation of HUVECs. **D.** Shift in PI distribution of early upregulated genes in TNF-alpha-stimulated IMR90 cells. ***, $p<0.001$, Mann-Whitney U test

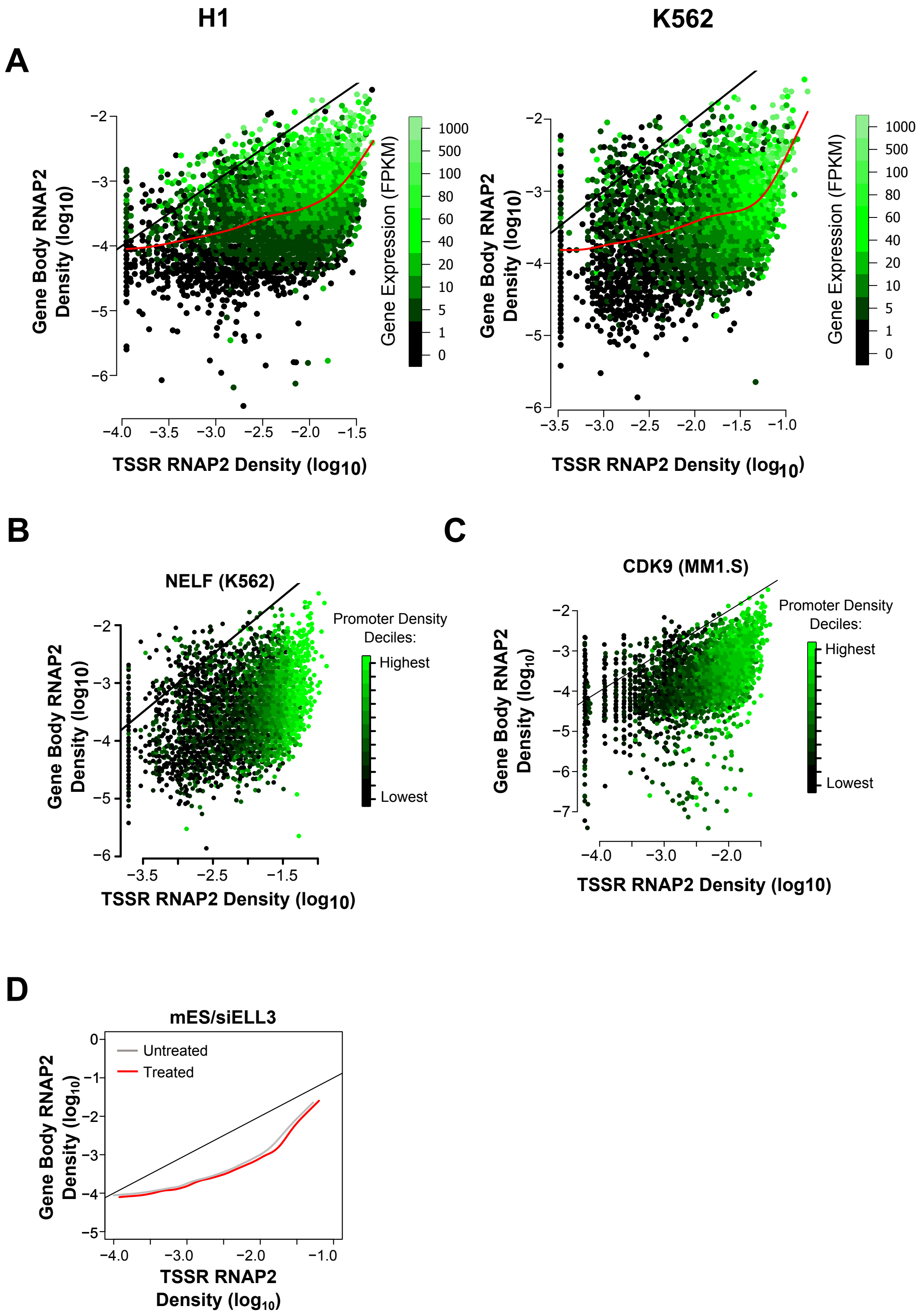

Supplementary Figure 8. Relationship of gene expression to TSSR and gene body RNAP2 density and to PI. A. Visualization of data from H1 and K562 cells. **B-C.** CDK9 and NELF occupancy of genes as a function of their TSSR and gene body RNAP2 densities. Both CDK9 and NELF were most enriched at genes with high TSSR RNAP2 density. CDK9 nor NELF enrichment were not strongly correlated with either gene body RNAP2 density or with PI. **D.** siRNA knockdown of ELL3 in murine ES cells had minimal effect on the TSSR-gene body RNAP2 density trend line (black line is where PI = 1).

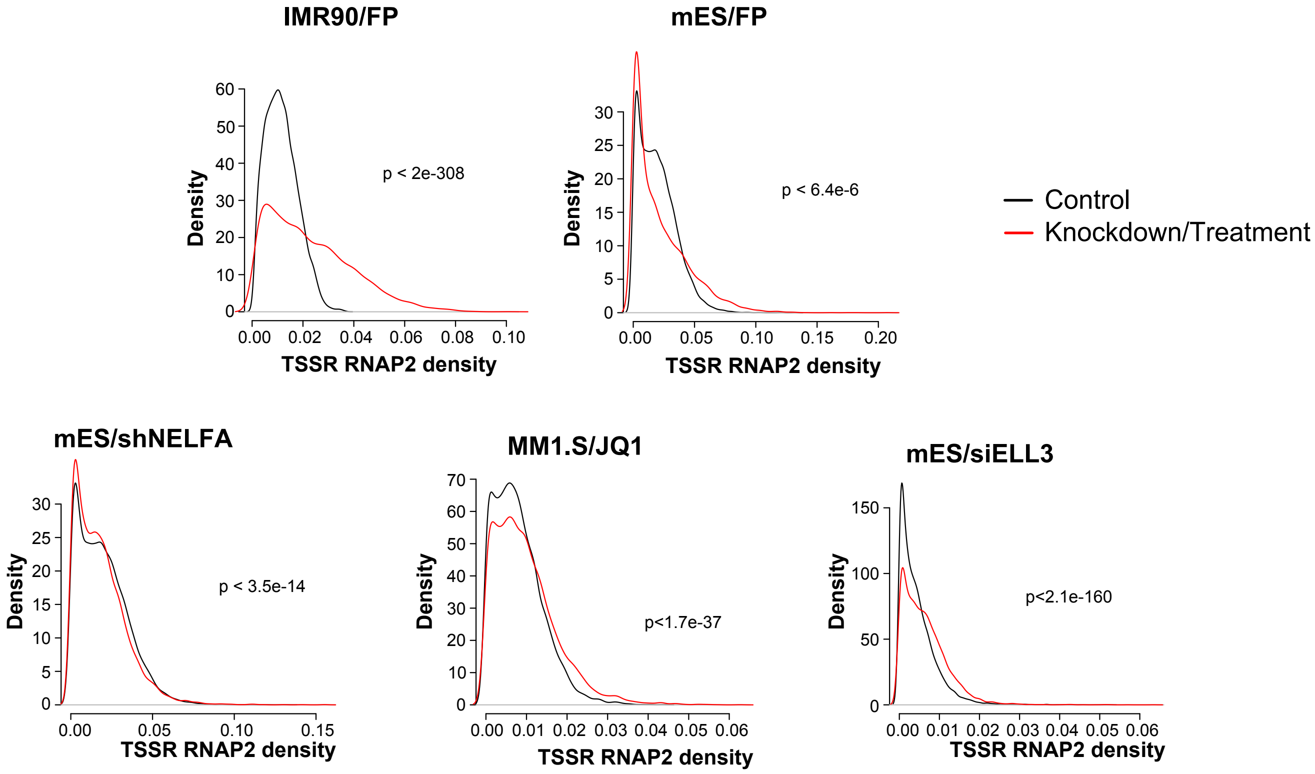

Supplementary Figure 9. The Inflection point is not driven by a limit to the level of initiating RNAP2. We compared the kernel-smoothed density distributions of TSSR RNAP2 density (as a proxy for initiated RNAP2) to see if the inflection point may be driven by a limit to how much RNAP2 can initiate. Especially in the FP-treated samples, we generally saw that the distribution of TSSR density was not static and extended to higher TSSR RNAP2 density. Moreover, for treatments expected to reduce RNAP2 pausing release (FP,JQ1,and siELL3), we observed a rightward shift in the TSSR density distribution compared to control, suggesting that it is possible to induce more RNAP2 occupancy near the TSS. These data indicate that the level of initiating RNAP2 is not saturated in control cells.

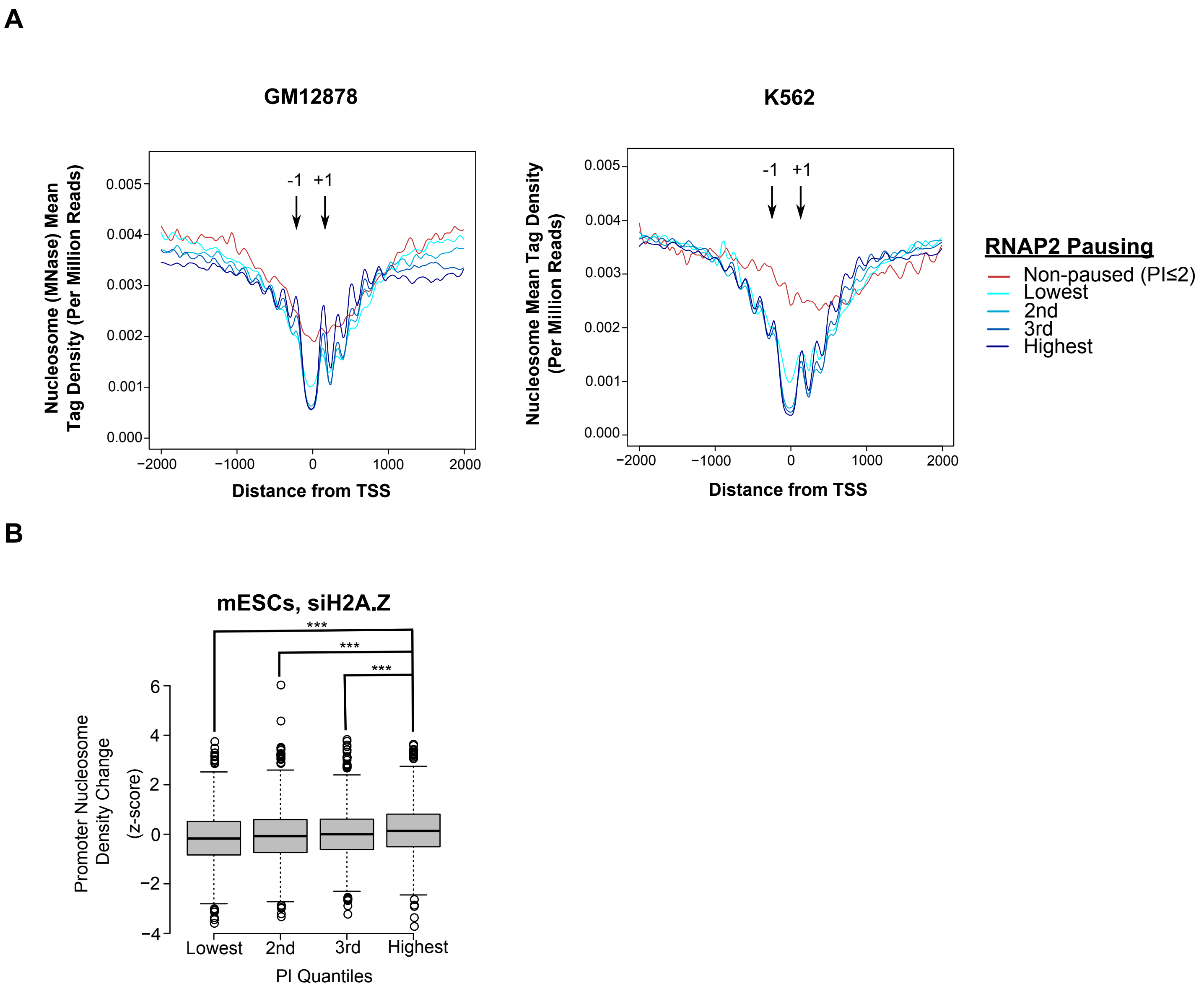

Supplementary Fig 10. Nucleosome positioning and RNAP2 pausing. A. Microccocal-digested (MNase) nucleosome profiles near TSSs were stratified by extent of gene pausing. Genes with significant paused RNAP2 were marked by greater nucleosome depeletion near TSS, especially for the top three quartiles of genes by PI. Despite overall nucleosome depletion near TSS, these genes maintained strong +1 and -1 nucleosomes (arrows). **B.** MNase nucleosome density change at the promoter gradually (where the densities were converted into z-scores, comparions made with the Mann-Whitney U test, *** $p < .001$) increased gradually with increasing PI upon H2A.Z knockdown in mES cells.

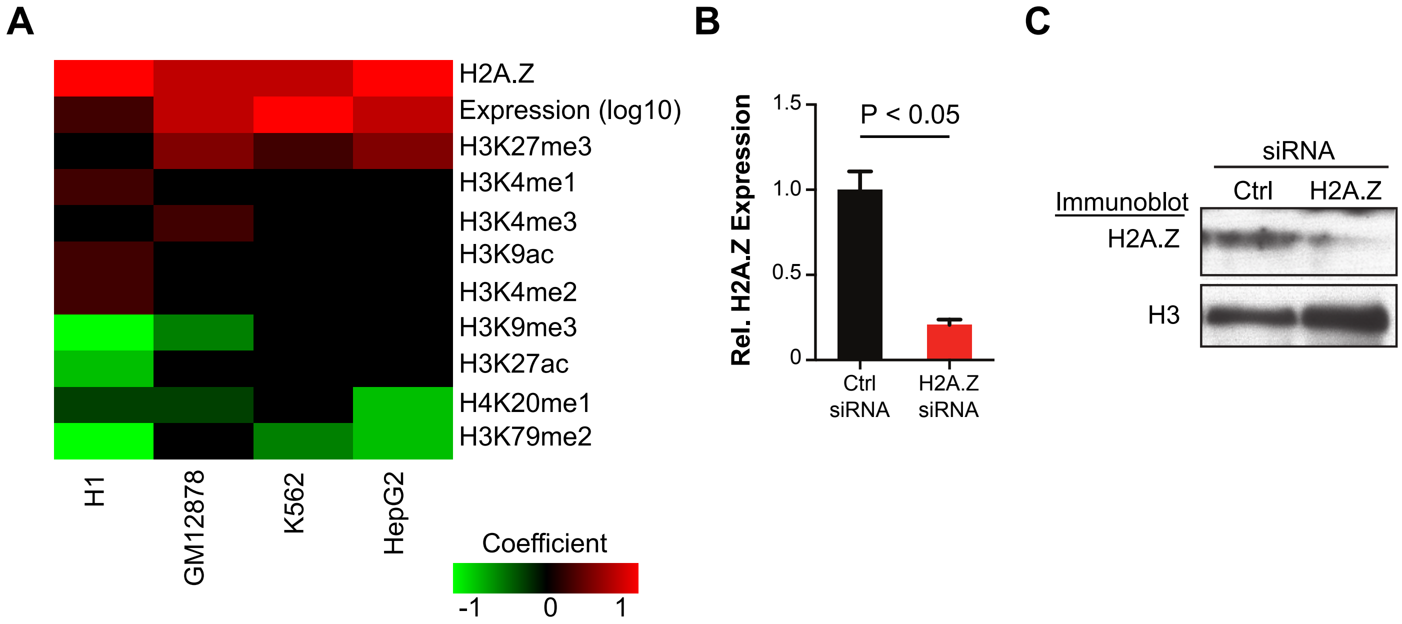

Supplementary Figure 11. Chromatin features and RNAP2 Pausing (in support of Figure 5). A. We performed a similar analysis of the correlation between promoter chromatin features and PI as in Figure 5A, but in addition included gene expression as an additional feature of the model (see Methods)**.** Even when including gene expression as a potential co-founding variable in the model, H2A.Z deposition at the TSS still positively correlated with a gene's PI across all four cell types. **B-C.** siRNA knockdown of H2A.Z. MCF-7 cells were treated with control or H2A.Z siRNA. mRNA (B) or protein levels (C) were measured by qRTPCR or western blotting, respectively.
